# Mitochondrial Genomes Suggest Rapid Evolution of Dwarf California Channel Islands Foxes (*Urocyon littoralis*)

**DOI:** 10.1371/journal.pone.0118240

**Published:** 2015-02-25

**Authors:** Courtney A. Hofman, Torben C. Rick, Melissa T. R. Hawkins, W. Chris Funk, Katherine Ralls, Christina L. Boser, Paul W. Collins, Tim Coonan, Julie L. King, Scott A. Morrison, Seth D. Newsome, T. Scott Sillett, Robert C. Fleischer, Jesus E. Maldonado

**Affiliations:** 1 Department of Anthropology, University of Maryland, College Park, Maryland, United States of America; 2 Program in Human Ecology and Archaeobiology, Department of Anthropology, National Museum of Natural History, Smithsonian Institution, Washington, District of Columbia, United States of America; 3 Center for Conservation and Evolutionary Genetics, Smithsonian Conservation Biology Institute, National Zoological Park, Washington, District of Columbia, United States of America; 4 Department of Vertebrate Zoology, National Museum of Natural History, Smithsonian Institution, Washington, District of Columbia, United States of America; 5 Department of Biology, Graduate Degree Program in Ecology, Colorado State University, Fort Collins, Colorado, United States of America; 6 The Nature Conservancy, San Francisco, California, United States of America; 7 Department of Vertebrate Zoology, Santa Barbara Museum of Natural History, Santa Barbara, California, United States of America; 8 National Park Service, Channel Islands National Park, Ventura, California, United States of America; 9 Catalina Island Conservancy, Avalon, California, United States of America; 10 Department of Biology, University of New Mexico, Albuquerque, New Mexico, United States of America; 11 Migratory Bird Center, Smithsonian Conservation Biology Institute, National Zoological Park, Washington, District of Columbia, United States of America; University of the Sunshine Coast, AUSTRALIA

## Abstract

Island endemics are typically differentiated from their mainland progenitors in behavior, morphology, and genetics, often resulting from long-term evolutionary change. To examine mechanisms for the origins of island endemism, we present a phylogeographic analysis of whole mitochondrial genomes from the endangered island fox (*Urocyon littoralis*), endemic to California’s Channel Islands, and mainland gray foxes (*U. cinereoargenteus*). Previous genetic studies suggested that foxes first appeared on the islands >16,000 years ago, before human arrival (~13,000 cal BP), while archaeological and paleontological data supported a colonization >7000 cal BP. Our results are consistent with initial fox colonization of the northern islands probably by rafting or human introduction ~9200–7100 years ago, followed quickly by human translocation of foxes from the northern to southern Channel Islands. Mitogenomes indicate that island foxes are monophyletic and most closely related to gray foxes from northern California that likely experienced a Holocene climate-induced range shift. Our data document rapid morphological evolution of island foxes (in ~2000 years or less). Despite evidence for bottlenecks, island foxes have generated and maintained multiple mitochondrial haplotypes. This study highlights the intertwined evolutionary history of island foxes and humans, and illustrates a new approach for investigating the evolutionary histories of other island endemics.

## Introduction

The origin of island endemism has long been an important topic in biogeography[1–4] and has implications for species management and conservation. Small populations of island endemic taxa are often at risk of extirpation or extinction due to their reduced genetic diversity and increased susceptibility to genetic drift, disease, and climate change, especially in conjunction with over-exploitation, habitat loss, and predation or competition from invasive species [4–7]. Island taxa typically experience substantial morphological and behavioral changes following dispersal and a period of isolation from the mainland [1]. Stepping-stone and other models have been proposed for the natural dispersal of a variety of taxa, but lessons from the invasive species pandemic of recent centuries suggest that ancient human introductions also may have been important dispersal mechanisms [8,9]. Because the evolution of many island taxa have been influenced by a combination of natural and anthropogenic dispersal events, distinguishing between these mechanisms requires archaeological, paleontological, and genetic data [8–10]. Understanding how island taxa evolved and adapted to their new environments can also improve our ability to manage island endemics in the face of rapid environmental change.

To investigate the mechanisms that generate island endemism, we studied the origins and evolution of the island fox (*Urocyon littoralis;*1–3kg), a diminutive canid endemic to six of California’s Channel Islands, and a congener of the gray fox (*U*. *cinereoargenteus;* 3–7 kg) found throughout mainland North America. The island fox is the largest endemic post-Pleistocene land mammal and a top predator on the Channel Islands. The recovery of fox populations on several islands following collapses due to predation and disease is among the great success stories of island restoration ecology [11–13]. Previous genetic research of modern island foxes and chronological and distribution analysis of island fox remains from paleontological and archaeological contexts posed different hypotheses about fox origins on the Channel Islands [14–22]. In one model, island foxes diverged from California gray foxes >16,000 years ago (prior to the arrival of humans) after rafting to the northern islands and were subsequently moved to the southern islands >5000 years ago by Native Americans [14–17]. However, the fox remains used to support a Pleistocene divergence as much as 40,000 years ago were recently dated via Accelerator Mass Spectrometry (AMS) ^14^C to 1480–1280 cal BP (calibrated calendar years before present) [21]. The earliest AMS dates for island foxes are now only ~ 7000 cal BP, some 6,000 years after people first arrived on the Channel Islands at ~13,000 cal BP [23]. These data raise the possibility that island foxes may have arrived on the islands later than previously thought, diverged very recently, and undergone rapid evolution.

**Fig 1:**
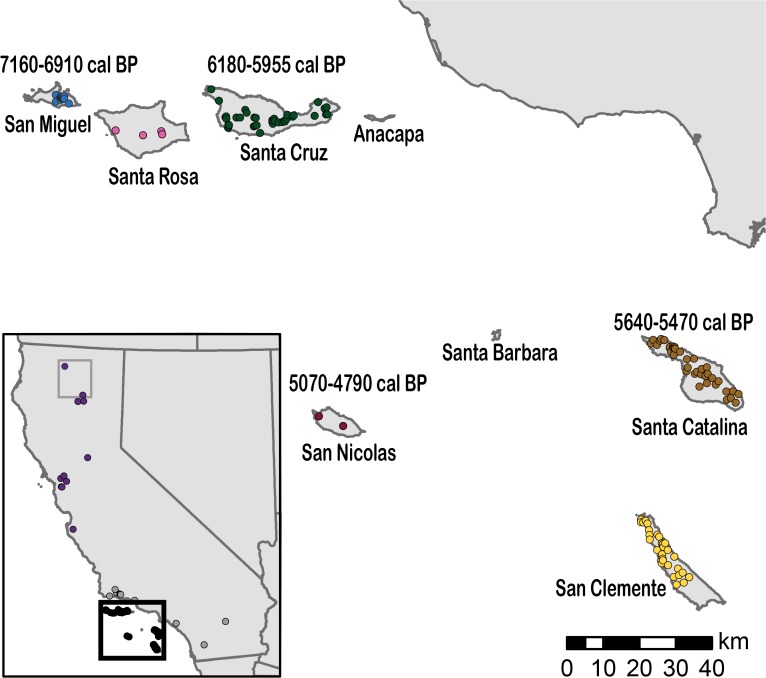
Sampled Localities of Island and Mainland Foxes. Mainland foxes were sampled from northern and southern California and island foxes were sampled from every island where they occur. The earliest directly AMS ^14^C dated island foxes are indicated. The two gray foxes most closely related to the island fox lineage in this study are enclosed in the gray box on the inset map.

The eight California Channel Islands are divided into northern and southern groups situated 20 to 98 km offshore (Fig. 1). The islands have an archaeological record spanning ~13,000 years, one of the earliest coastal human sequences in North America [23]. Sea level models suggest that the islands were larger and closer to the mainland and each other during the terminal Pleistocene and early Holocene but, throughout the Quaternary, they were always separated from the mainland by a watergap of at least 7 km [23,24]. During the last glacial maximum, the northern islands (Anacapa, Santa Cruz, Santa Rosa, and San Miguel) formed a super-island called Santarosae that began to separate as sea level rose ~11,000 cal BP, and were completely separated by 9000 cal BP [24]. Although the southern islands (San Clemente, San Nicolas, Santa Barbara, and Santa Catalina) increased in size during Quaternary glacial periods. they are more widely dispersed and were never connected to each other or the mainland during this time. These fluctuations in island size and distance from each other and the mainland influenced colonization and extinction rates of island taxa. As a result, the Channel Islands have several rare endemics, lower species diversity than the mainland, and only 10 known species of terrestrial mammals (excluding bats) [25].

Decades of research on island fox morphology and interactions with humans have documented their smaller size relative to mainland gray foxes, the occurrence of intentional island fox burials, and the use of fox pelts by Native Americans [18–22], but their evolutionary history has remained unresolved. Previous genetic research, using microsatellites, mtDNA restriction digests, allozymes, MHC, and DNA fingerprinting, primarily focused on island fox genetic variation and divergence between islands [14–17]. A recent phylogenetic analysis of the Canidae suggests that island and gray foxes are sister taxa [26], and earlier genetic analyses assigned gray foxes as an outgroup to island foxes [14,15]. To date, the only phylogeographic study of gray foxes was conducted on populations in the eastern United States [27]. However, the phylogeographic patterns between eastern, western, and island *Urocyon* populations had not been determined.

Mitochondrial DNA is a powerful marker for dating mammalian divergences and mitogenomes and has been used to examine both phylogeographic and evolutionary relationships in wild and domesticated animals, including canids [28–32]. Here, we present the first application of high throughput sequencing (HTS) of whole mitochondrial genomes (mitogenomes) to evaluate the phylogeographic patterns of differentiation between island and mainland gray fox populations. Our goal is to reconstruct the evolutionary history of island and gray foxes and to unravel their patterns of colonization into the Channel Islands. We address the following questions: 1) When did foxes reach the Channel Islands? 2) How have human activities and climatic changes influenced their genetic diversity and the geographic distribution of genetic lineages? 3) How can these data inform our understanding of the processes leading to island endemism?

## Results

### Genetic Variability

**Fig 2:**
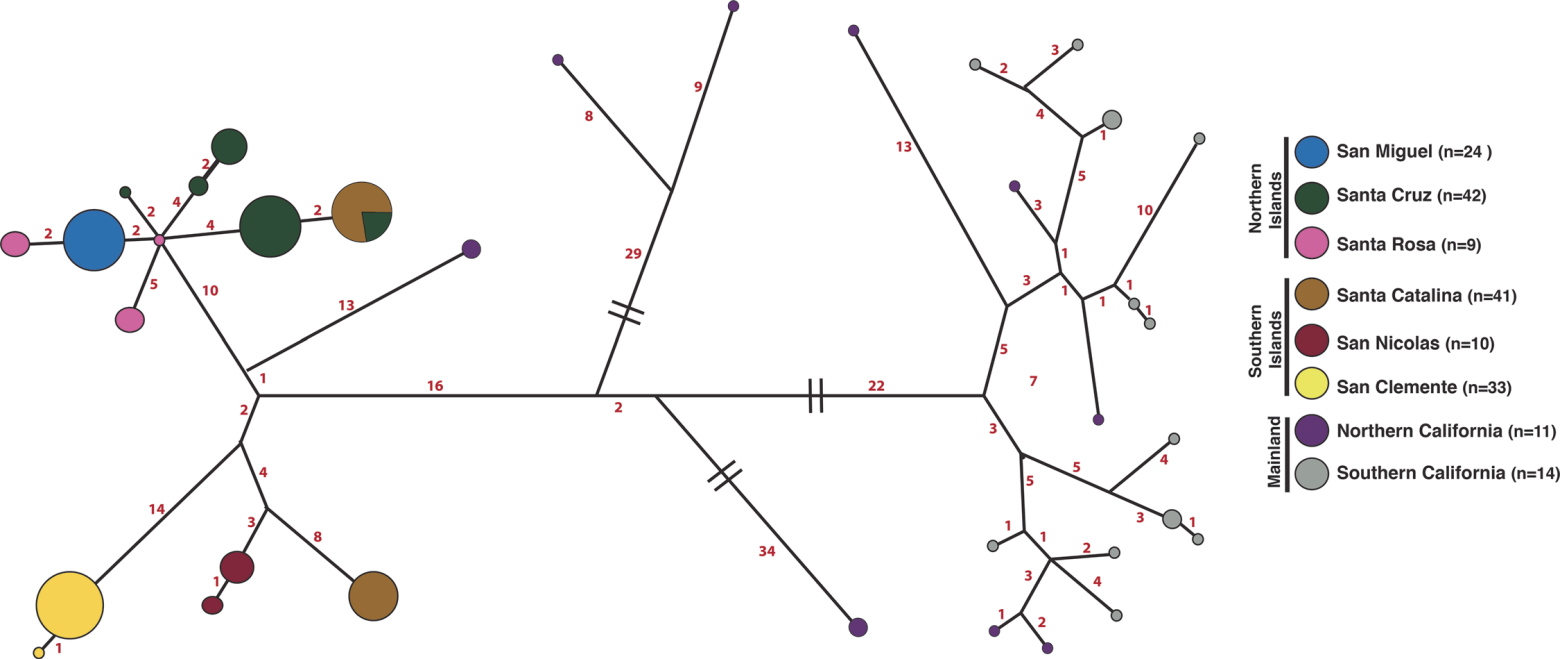
Median-Joining Network of Island and Mainland Mitochondrial DNA. A Median joining network using the variable sites of the mitochondrial genome was generated in the program Network v.4.612. The size of the circles and branch lengths are proportional to number of individuals represented and the number mutations between haplotypes (red), respectively. Hash marks indicate shortened branches. Santa Catalina and Santa Cruz are the only islands that share a haplotype, which is more closely related to the northern island haplotypes than the southern.

We sequenced complete mitogenomes from 159 modern (2007–2013) island foxes from the six islands in their current range and 25 gray foxes (1996–2013) from across California (Fig. 1 and Table 1). De Novo assembly revealed a 16,718 bp gray fox mitogenome with a mean read depth of 171x. However, a short fragment (248 bp) of the D-loop was excluded from all subsequent analyses due to problems with assembly and mapping of repetitive regions, leaving an alignment of 16,470 bp for all foxes. Mean read depth for all samples ranged from 33 to 7898x (std. deviation of 16 and 204, respectively) (S1 Table). We excluded samples with conflicting or incomplete mitogenomes from analysis (n = 16), yielding 185 complete mitogenomes. The mitogenome sequences revealed a total of 35 haplotypes with 14 found exclusively on the islands and 21 found only in mainland California. Haplotype and nucleotide diversity in the island populations was markedly lower than in the mainland populations, with only one to five haplotypes per island. The northern islands had nine closely related haplotypes while the southern islands had five haplotypes that were more distant from each other (Fig. 2). All of the islands had a least one private haplotype, but 19 of 41 Santa Catalina foxes (southern island) shared a haplotype with Santa Cruz foxes (northern island). No other islands shared a haplotype, although San Clemente and San Nicolas each had two haplotypes that were separated by a single base pair. Independent analyses of two of the most widely used mtDNA genes in mammalian phylogeography, cytochrome b and D-loop, recovered only 15 and 20 haplotypes, respectively, instead of the 35 haplotypes recovered by sequencing whole mitogenomes (S2 Table). These two genes represent a reduced portion of the genetic variation of island and mainland foxes as demonstrated by single-gene median joining networks (S1 Fig.); thus, they provide insufficient resolution to accurately investigate the colonization patterns from the mainland. Across the entire mitogenome, island foxes showed a three-fold reduction in the proportion of variable sites compared to mainland gray foxes (S2 Table).

**Table 1:** Mitogenome Haplotype Summary.

Locality	N	Haplotypes	Haplotype Diversity	Nucleotide Diversity
San Miguel	24	1	0	0
Santa Rosa	9	3	0.667	0.0003
Santa Cruz	42	5*	0.6	0.00024
Santa Catalina	41	2*	0.51	0.00099
San Nicolas	10	2	0.356	0.00002
San Clemente	33	2	0.061	0
***All Islands***	***159***	***14***	***0*.*865***	***0*.*00113***
Northern California	11	9	0.964	0.00342
Southern California	14	12	0.978	0.00125
***Mainland***	***25***	***21***	***0*.*987***	***0*.*00248***
Virginia	1	1	-	-
***Total***	***185***	***36***	***0*.*9***	***0*.*00198***

* Santa Catalina and Santa Cruz are the only islands that share a haplotype.

To evaluate the patterns of selective pressures acting on mainland and island fox mitogenomes, we estimated the proportion of nonsynonymous (dN) and synonymous variable (dS) sites throughout all of the protein-coding genes (Table 2). Although elevated dN/dS is a common metric for evidence of selection when comparing single genes, dN/dS values are much lower for signatures of selection across the entire mitogenome [33]. Using the SLAC algorithm implemented in HyPhy [34], the island group had a substantially higher mean dN/dS ratio (0.40) than mainland only (0.10), suggesting a signal of positive selection or the relaxation of selective forces associated with a decline in effective population size [35].

**Table 2:** Nonsynonymous and Synonymous Substitutions.

		Non-synonymous	Synonymous	Mean dN/dS
Island Only	Number of Sites	8500.49	2785.51	0.40
	Number of Variable Sites	22	27	
	Proportion of Varied Sites	0.0026	0.0097	
Mainland Only	Number of Sites	8497.41	2788.59	0.10
	Number of Variable Sites	29	148	
	Proportion of Varied Sites	0.0034	0.0531	

### Chronological analysis

Archaeological and paleontological research has identified more than 100 fox bones from more than 35 different archaeological and subfossil sites across the archipelago. Many of the bones have relatively secure associated radiocarbon ages; all of which are more recent than 7200 cal BP [18,19,21]. Morphological analysis of these ancient bone samples indicate the characteristic island fox morphology, even in the oldest sites [19]. To investigate the antiquity of foxes on the Channel Islands and improve radiocarbon chronologies, we obtained three new AMS radiocarbon dates of island fox bones from the potentially earliest archaeological contexts to complement previously published AMS dates. This yielded a total of nine directly radiocarbon dated ancient fox bones (S3 Table). The earliest fox AMS radiocarbon estimates date to 7160–6910 cal BP on the northern islands (San Miguel) and to 5640–5470 cal BP on the southern islands (Santa Catalina). We found no fossil or archaeological evidence of foxes on any of the Channel Islands prior to 7160 cal BP. Our AMS dated samples show phenotypic characteristics of island foxes (i.e. smaller size), though these bones are fragmentary. Fox bones with the characteristic gray fox morphology have been recovered from relatively few archaeological sites on the coastal mainland; none have been recovered on the Channel Islands. Similarly, no small fox has been identified in mainland assemblages.

**Fig 3:**
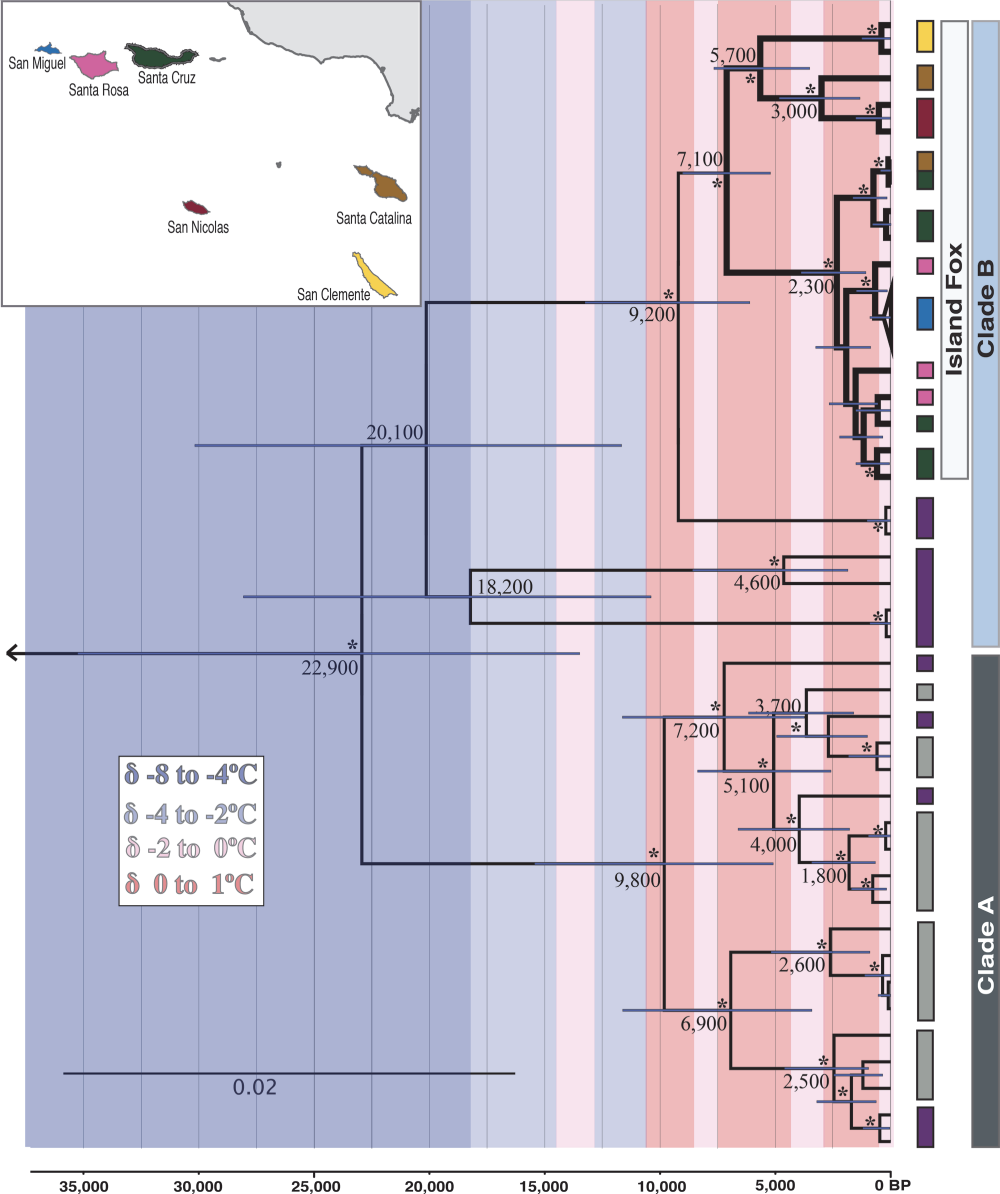
Bayesian phylogeny of mitogenomes of island and mainland foxes. The northern Channel Island foxes (San Miguel, Santa Rosa and Santa Cruz) diverged from the southern island foxes (Santa Catalina, San Clemente, and San Nicolas) ~7100 years BP (lineage bolded). Northern California (purple) and southern California (gray) foxes show patterns of climate-induced expansion. Divergence dates were calculated in BEAST v1.7.5 with node bars indicating height 95% highest posterior density. Nodes with * indicate greater than 0.99 posterior probability. Global surface temperature is overlaid in δ°C to show how climatic events may have impacted fox diversity [55–57]. Local temperature curves were not used due to the geographic distance sampled and the short time scale of local curves.

Bayesian phylogenetic analysis in BEAST [36] of all unique mainland and island haplotypes (Fig. 3) used the earliest island fox radiocarbon date as a prior estimate for the coalescence of all island fox lineages. This tree yielded the same topology as a maximum likelihood analysis with *Vulpes* and *Canis* outgroups; both trees had strong support (S2 Fig.). Our phylogenetic analysis (Fig. 3) revealed that fox haplotypes fell in two well-supported and divergent clades (Clades A & B). However, gray foxes from southern and northern California did not show a strong pattern of contemporary phylogeographic structure, and their haplotypes did not form reciprocally monophyletic clades. Haplotypes in clade A included all individuals sampled in southern California, plus some haplotypes from northern California. Our estimates of divergence suggest that clades A and B diverged approximately 22,900 years ago (95% Highest Posterior Density [HPD]: 35,300–13,500). Remarkably, island fox haplotypes formed a monophyletic clade nested within clade B, rather than with foxes from southern California, closest to the Channel Islands. Among the gray foxes sampled, the haplotypes that were more closely related to the island fox were from Lassen/Shasta counties in northern California. Our estimates suggest that gray and island foxes diverged ~9200 years ago (95% HPD: 13,300–6100) and that the divergence between foxes on the northern and southern islands likely occurred ~7100 years ago (95% HPD: 9000–5200). Collectively, the radiocarbon, archaeological, paleontological, and genetic evidence roughly support the ~9200 and ~7100 estimates. Note that central California is our weakest sampling area. Future studies should focus on obtaining samples in this region.

## Discussion

### Gray and Island Fox Phylogeography

A broad scale analysis of the evolutionary relationships of *Urocyon* lineages should be conducted because in this study, we found that eastern and western gray fox populations are more deeply diverged than the two currently recognized species of California *Urocyon* (S2 Fig.). Furthermore, the island fox mitogenomic diversity (Fig. 3) is nested within that of the northern California mainland gray foxes. Adaptive divergence between island and mainland populations is likely and nuclear data will be important for re-examining species and sub-specific designations.

We propose the following hypothesis for the phylogeographic pattern observed in clade A California mainland gray foxes: During the late Pleistocene, approximately 23,000 years ago (Fig. 3) populations of California foxes diverged into two well-supported clades (clade A and B). Glacial climatic fluctuations caused habitat changes, including the appearance of continental ice sheets as far south as Washington State [37], that may have caused range shifts in locally adapted gray fox populations, with foxes with clade B haplotypes existing as far south as southern California. The current northern range extension of gray foxes is well into northern Oregon. However, the climate-induced shifts in habitat that occurred during the late Pleistocene, may have influenced a southward shift in the distributional range of gray foxes. As the climate warmed during the Holocene, and suitable habitat expanded northward, gray fox population ranges shifted further north resulting in foxes with clade A haplotypes distributed as far north as Shasta County in northern California (Fig. 1). Niche modeling with finer-scaled sampling and further genetic analysis is needed to test our hypothesis. Interestingly, a similar climatic-induced, phylogeographic pattern was found in eastern gray foxes [27]. The historic expansion of gray foxes into northeastern US during the Medieval Climate Anomaly may have coincided with the expansion of eastern deciduous forests [27].

Island foxes form a monophyletic group within the northern California gray fox clade (Clade B; Fig. 3). Santa Catalina and Santa Cruz foxes share a haplotype (Fig. 2), suggesting a recent human introduction of foxes from Santa Cruz to Catalina. This scenario is supported by similarities between these islands in morphological and mtDNA restriction hybridization data collected before 1990 and the later fox population bottlenecks [15,20]. Santa Catalina was a center for trade between Native Americans on the mainland and the southern and northern Channel Islands, with evidence for exchange of a variety of goods including soapstone artifacts from Santa Catalina quarries [20,38].

Previous analyses using mtDNA RFLP’s identified more mtDNA haplotypes than detected by our whole mitogenome (WMG) sequencing analysis on San Miguel (2 RFLP: 1 WMG genotypes) and Santa Catalina (3 RFLP: 2 WMG genotypes), suggesting a possible loss of mtDNA diversity on these islands as a result of population bottlenecks over the past 25 years [15]. MtDNA RFLP data from Santa Cruz, Santa Rosa, San Nicolas, and San Clemente identified the same number or one fewer haplotypes than whole mitogenomes. Sample size does not account for this pattern as mitogenome sample sizes were larger on all islands except Santa Rosa and San Nicolas. San Nicolas island foxes were found to be monomorphic at 18 microsatellite loci but showed higher heterozygosity in MHC loci [17]. We identified two pairs of closely related haplotypes on each of the two most remote islands in the archipelago (San Nicolas and San Clemente), which indicates *in situ* evolution of island fox genetic variation. Furthermore, the fox populations on San Nicolas and San Clemente islands did not undergo as severe population crashes as the other island fox populations experienced. This might explain the presence of additional haplotypes, especially in comparison to the population crashes and the potential loss of genetic diversity on San Miguel and Santa Catalina.

We find evidence of discrete island-specific matrilines and microsatellite loci detected a strong signal of population genetic structure in island foxes [14]. Mitogenomes can recover patterns at greater time depths than microsatellites, which are likely to reflect recent and dramatic changes in nuclear allelic frequencies caused by genetic drift [14,17]. Strong population differentiation among microsatellites can occur in just decades [39], so it is plausible to detect both fine-scale genetic structure with microsatellites and low levels of divergence between mitochondrial lineages.

### Origins of the Island Fox

The diversification events influencing island and mainland gray foxes closely correspond to climatic fluctuations, particularly the shift from fully glacial to non-glacial conditions during the past 20,000 years. The arrival of foxes on the Channel Islands could have occurred when the range of clade B gray foxes shifted into southern California. Our divergence date estimates for the island fox clade itself, ~9200 years ago, as Santarosae separated into four distinct islands and for the southern island fox lineage, ~7100 years ago, correspond well with the earliest AMS dated island fox bones (~7100 cal BP for northern and ~5640 cal BP for the southern islands). All of these data imply a Holocene colonization. However, the fact that we detected only 13–14 base pair substitutions between the closest mainland gray fox lineage and the northern/southern island fox ancestor suggests that the date of divergence for the island fox clade could be more recent than 9200 years ago.

Radiocarbon dates of nine island fox bones suggest that the northern Channel Islands were colonized first, followed by dispersal to the southern islands. Island foxes show greater mitogenome diversity in the northern islands with nine haplotypes that differ from 2–5 base pairs from the central node of the star-shaped radiation (Fig. 2). This pattern is strikingly different from the southern islands, where San Clemente and San Nicolas each have two haplotypes just a single base pair away from each other. The greater genetic diversity and radial haplotype pattern in the northern islands may have taken longer to occur than the single mutational step found in the southern islands. However, a bottleneck in the northern islands followed by population growth could also generate this pattern. The mitogenomic data are consistent with the archaeological evidence that supports the hypothesis that foxes dispersed from northern to southern islands but more data are needed to confirm a northern to southern island dispersal.

Island foxes are not strong overwater dispersers and it is extremely unlikely they reached the more remote southern islands without human intervention [18,19,40]. Therefore, the substantial divergence between southern and northern island lineages in network and phylogenetic analyses implies that foxes were moved to the southern islands soon after they arrived on the northern islands. Our results suggest that southern island lineages diverged from each other 5470–5640 cal BP to 5700 years ago (95% HPD: 7700–3500) based on radiocarbon and genetic date estimates, respectively. Although the estimates obtained by radiocarbon dating may more accurately represent the arrival date rather than a divergence date estimate provided by the mitogenomic data, the radiocarbon dates and divergence estimates are very similar. Previous estimates using microsatellites and an ultrametric UPGMA bootstrap consensus tree based on (δμ)^2^ genetic distances with low support calculated the divergence of southern lineages (excluding San Nicolas) to be 5539 years ago and 12,000 years ago for the island fox lineage as a whole [14]. These earlier estimates were calibrated using a presumed initial fox introduction date of 16,000 years ago, which is no longer supported by AMS radiocarbon dates or other evidence.

Our genetic results also indicate that divergence within northern island lineages occurred more recently than our radiocarbon dates. The divergence estimate of 2300 years ago for all northern island lineages may be evidence of a severe bottleneck that either happened before 2300 years ago and resulted in the reduction of ancient mtDNA lineages, or an indication that northern island fox populations were panmictic due to human intervention before 2300 years ago. Estimates of divergence using microsatellites between San Miguel and Santa Rosa, yielded a date of 2079 years ago, which is comparable to our results [14]. Although in the same analysis, the Santa Cruz lineage shows a deeper divergence (7522 years ago), which is very close to our estimates of the entire island fox lineage. However, this node in the microsatellite analysis is not well supported with a bootstrap value of 49.6%. Interestingly, the most recent 3500 years of prehistory was a time of Native American population growth and sociopolitical changes, perhaps increased human-induced fire, as well as a period of climatic instability [41,42], both of which could have affected contemporary levels of mtDNA diversity. Present day population dynamics and climatic events may have similar effects as past climate instability.

The precise mode of fox colonization of the northern islands remains unclear, but our genetic and radiocarbon data indicate that this event occurred well after human colonization (~13,000 cal BP). Two hypotheses provide plausible colonization scenarios. First, a single pregnant fox or even a few closely related foxes, rafted to the northern islands while Santarosae was splitting into four separate islands (or just after they had separated) between 10,000–9000 cal BP or later. Gene flow between the islands decreased and populations became isolated, giving rise to island-specific lineages. Humans later transported island foxes to San Clemente, Catalina, and San Nicolas islands. The second hypothesis is that Native Americans introduced foxes coincident with the breakup of Santarosae. Similar to the first hypothesis, island foxes were then moved to the southern islands. We cannot reject either hypothesis, but both involve prehistoric human intervention and translocation. The ancient gray fox lineage that gave rise to all island foxes appears to be unsampled and may be rare or extinct in the extant gray fox populations. Ancient DNA analysis of archeological samples are needed to further distinguish between these different scenarios, because prehistoric and recent bottlenecks may confound the patterns that we obtained from contemporary fox samples.

### Endemism and Rapid Evolution

Our data suggest that the rapid evolution of unique behavioral and morphological features associated with island endemism in *U*. *littoralis* is the result of close interaction with humans as well as island evolutionary pressures and climate change. The small size and tameness of island foxes are traits often linked to insular evolutionary pressures [1]. Island fox dwarfism may have occurred rapidly (2 millennia or less) because the earliest fox bones (~7100 cal BP) were already small in size. Alternatively, island foxes may have originated from small mainland gray foxes, but we have no evidence that such a mainland population existed. Island dwarfism is thought to result from intense natural selection caused by evolutionary pressures of living on islands (e.g., increased competition for limited resources) [43]. Rapid morphological change has occurred many times in mammalian evolutionary history [44], with and without human intervention, including domestic dogs [45] and silver foxes (tamed from wild Russian red foxes (*Vulpes vulpes*) [46–48].

We detected limited evidence for natural or artificial selection in island fox mitogenomes. A comparison of mitogenomes from 16 pairs of domestic animals and their wild progenitors revealed no consistent patterns between divergence date, dN/dS, and branch length [33], indicating that human-mediated selective pressures did not have consistent mitogenomic effects. Changes to dn/ds in some of these domestic/wild animal comparisons may be the result of reductions in effective population size leading to relaxation in selective forces. However, the average difference between dN/dS was only 0.0342 in these domesticated/wild comparisons [33], whereas island foxes (0.40) have almost a fourfold greater dN/dS ratio than California mainland foxes (0.10). This elevated dN/dS ratio in island foxes may be due to reduced effective population size during colonization, bottlenecks, or artificial selection.

Humans have had close associations with island foxes for thousands of years, which could have exerted selective pressure for a small, tame fox phenotype, Native Americans practiced intentional island fox burials [18,19] and translocated island foxes to the southern islands early in the evolutionary history of *U*. *littoralis*. Anglo-American ranchers purportedly introduced a few island foxes from Santa Catalina to San Clemente in 1875 [49] and island foxes were kept as pets in the 20^th^ century [19,49]. Clearly, island fox evolutionary history has been intertwined with humans across the entire timespan of Native American, Euro-American ranching, and modern conservation management eras.

Island-specific mitochondrial lineages and *in situ* evolution also suggest that island foxes have undergone dramatic and rapid evolution over the past ~9–7000 years. Despite small population sizes and limited geographic distribution, island foxes have generated and maintained mitochondrial diversity, even with population reductions to only 15 individuals on some islands during the 1990s [11]. These island fox data, however, do not reveal the past diversity that may have been lost during recent or even historical bottlenecks [50,51]. Regardless of whether the original fox population arrived on the Channel Islands by natural or human dispersal, island foxes have adapted to and weathered dramatic environmental and cultural change for more than seven millennia.

This study provides a new approach to integrating archaeological, paleontological, and biological datasets to examine biogeographic patterns of wild animals and plants and the evolution of endemic and endangered species. Collaborative research teams of archaeologists, biologists, geneticists, and resource managers can generate new insights about the evolutionary histories of other endemic taxa. We expect such investigations of the deep histories of human-animal interactions to become increasingly important for understanding the relationships between people and the natural world and for guiding conservation decisions.

## Materials and Methods

### AMS Radiocarbon Dating

All ages derived from AMS radiocarbon dates are in calibrated calendar years (cal BP) unless otherwise noted (S1 Text and S3 Table).

### Mitogenome Sequencing

For this study we exclusively used tissue samples that had previously been deposited in several frozen tissue collections from recognized institutions including those housed at CCEG, at the Smithsonian Conservation Biology Institute, the Santa Barbara Museum of Natural History, the Catalina Island Conservancy, the Museum of Vertebrate Zoology at Berkley, the Colorado State University, the Nature Conservancy, the National Park Service collections (permit number CHIS-2012-SCI-0006) including the National Park Service Special Collection at Ambrose Monell Cryo Collection at the American Museum of Natural History (see S1 Table). No animals were trapped or sacrificed for the purposes of this study and therefore, a formal approval by an Institutional Animal Care and Use Committee was not necessary. Total genomic DNA was extracted in a PCR product-free extraction lab using DNeasy Blood and Tissue DNA kits (Qiagen). 185 whole mitogenomes were generated from 201 samples using three different library prep protocols and two sequencing methods, 454 and Illumina (S1 Text and S1 Table). The same haplotypes were recovered from both sequencing methods indicating no sequencing method bias.

Raw reads were trimmed, filtered and mapped with BWA v.0.7.4 to a gray fox reference that was assembled deNovo with Mira v3.4.0. Consensus sequences and coverage were calculated with SamTools v0.1.19 and all consensus sequences were aligned with Mafft v7.017. The aligned mitogenomes were visually examined and when a single island individual or an ambiguous base generated a unique haplotype, Sanger sequencing was conducted to verify the basecalls (S1 Text and S4 Table), which did not change except for two samples with conflicting haplotypes that was discarded from all analyses. Sequences have been deposited in GenBank (accession numbers KP128924- KP129108).

Summary analyses were completed in DNAsp v5.10.1, GenALEx v.6.5 and Arelquin v3.5. Network analysis was conducted on an alignment stripped of monomorphic sites using the median joining algorithm and default parameters of Network v.4.612. Selection analysis was completed using an alignment of coding genes (11,286 bp), and a neighbor-joining tree with the same topology as the Bayesian and ML tree. These datasets were uploaded to the HyPhy data server and six algorithms were used with the HKY85 model.

### Phylogenetic analyses

To examine phylogenetic relationships between island, California and eastern gray foxes, additional publically available mammal mitogenomes were obtained from GenBank and aligned to the fox dataset. The alignment was run through JModelTest v.2.1 and the GTR+I+ Γ model was used to run 1000 pseudobootstrap replicates of the maximium likelihood tree program Garli as implemented on the Lattice grid computing system [52,53].

To date the divergence between island and mainland foxes, Bayesian phylogenetic analysis was conducted in BEAST v.1.7.5. Each gene, as well as the entire alignment, were run through JModelTest v2.1 and PartitionFinder v1.1.1. Based on this analysis, no codon partitioning and empirical base frequencies were used with each gene fitting the HKY or the TN93 model. Both a lognormal relaxed and strict clock were tested with a coalescent of constant size. The earliest calibrated radiocarbon date was used as a prior estimate of the time to the most recent common ancestor for all island fox samples. An eastern gray fox sample was used as an outgroup as indicated by the maximum likelihood analysis. The eastern gray fox is deeply diverged from California foxes but this split must be younger than the oldest fossil date for *Urocyon* so we set the root length to the early Pliocene *Urocyon* fossil dating to 5.332–2.558 MYA [54].

All other priors were left to default settings and the MCMC was run in two independent runs of 100 million chains each, logging every 10,000 chains. An empty alignment was tested to sample for effects of the prior and the resulting poor posterior and prior ESS with values below 200 indicated that the priors were not strongly influencing the tree. Substitution rates were compared to other canids and mammals (see S1 Text) to examine how the radiocarbon date prior influenced divergence dates.

## Supporting Information

S1 Fig Network Analysis of cytochrome b and d-loop. Cytochrome b (1140 bp) only network (A) and d-loop (992 bp) only network (B) were generated from variable alignment sites. The size of the circles is proportional to the number of individuals represented by it. Neither cytochrome b nor d-loop had enough variants to detect all island-specific lineages.
(TIF)

S2 Fig Maximum Likelihood Tree of Island and Gray Foxes. Rooted tree generated in Garli with 1000 bootstrap replicates. Key nodes are shown with bootstrap support. Nodes that are not labeled may also have strong support. Eastern gray fox is basal to the California clade and there is strong support for Clade A and Clade B (Fig. 3).
(TIF)

S3 Fig Haplotype and Haplotype Diversity Correlate with Island Area. A positive correlation was identified between island area and the number of haplotypes recovered and haplotype diversity (Pearson’s r = 0.80 p-value = 0.03 and r = 0.77 p-value = 0.04, respectively).
(PDF)

S1 Sequences Mitogenome capture probe sequences. (FASTA)

S1 Table Sample and Coverage Information. (DOCX)

S2 Table Distribution of polymorphisms in island and gray fox mitogenomes. (DOCX)

S3 Table AMS Radiocarbon dates of island foxes. (DOCX)

S4 Table Primer Sequences. (DOCX)

S5 Table Distances between Mainland and Islands. (DOCX)

S1 Text Supplementary Materials and Methods. (DOCX)
