# Supplementary figures and images for: Mitochondrial Genomes Suggest Rapid Evolution of Dwarf California Channel Islands Foxes (Urocyon littoralis)
Source: PLoS One. 2015 Feb 25;10(2):e0118240. doi: 10.1371/journal.pone.0118240 (PMC4340941; doi:10.1371/journal.pone.0118240)

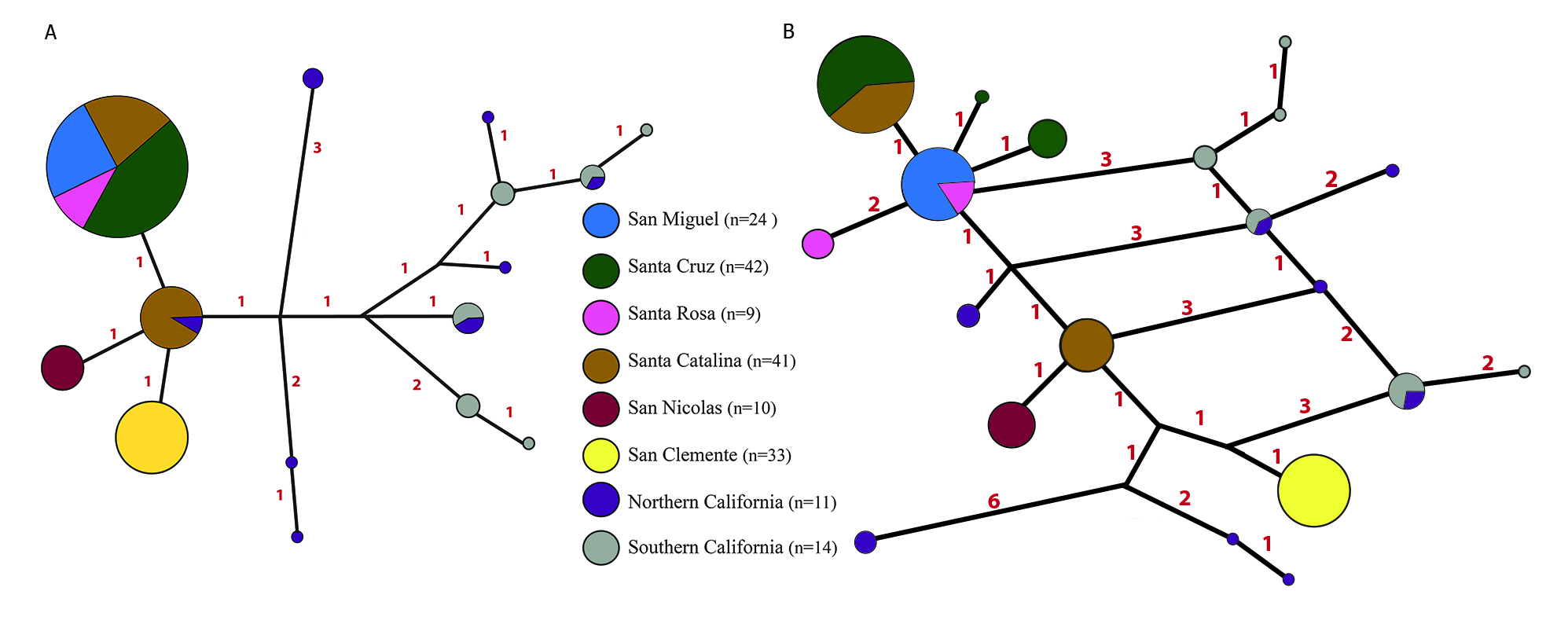

Supplement: S1 Fig — Cytochrome b (1140 bp) only network (A) and d-loop (992 bp) only network (B) were generated from variable alignment sites. The size of the circles is proportional to the number of individuals represented by it. Neither cytochrome b nor d-loop had enough variants to detect all island-specific lineages. (TIF) [file pone.0118240.s001.tif]

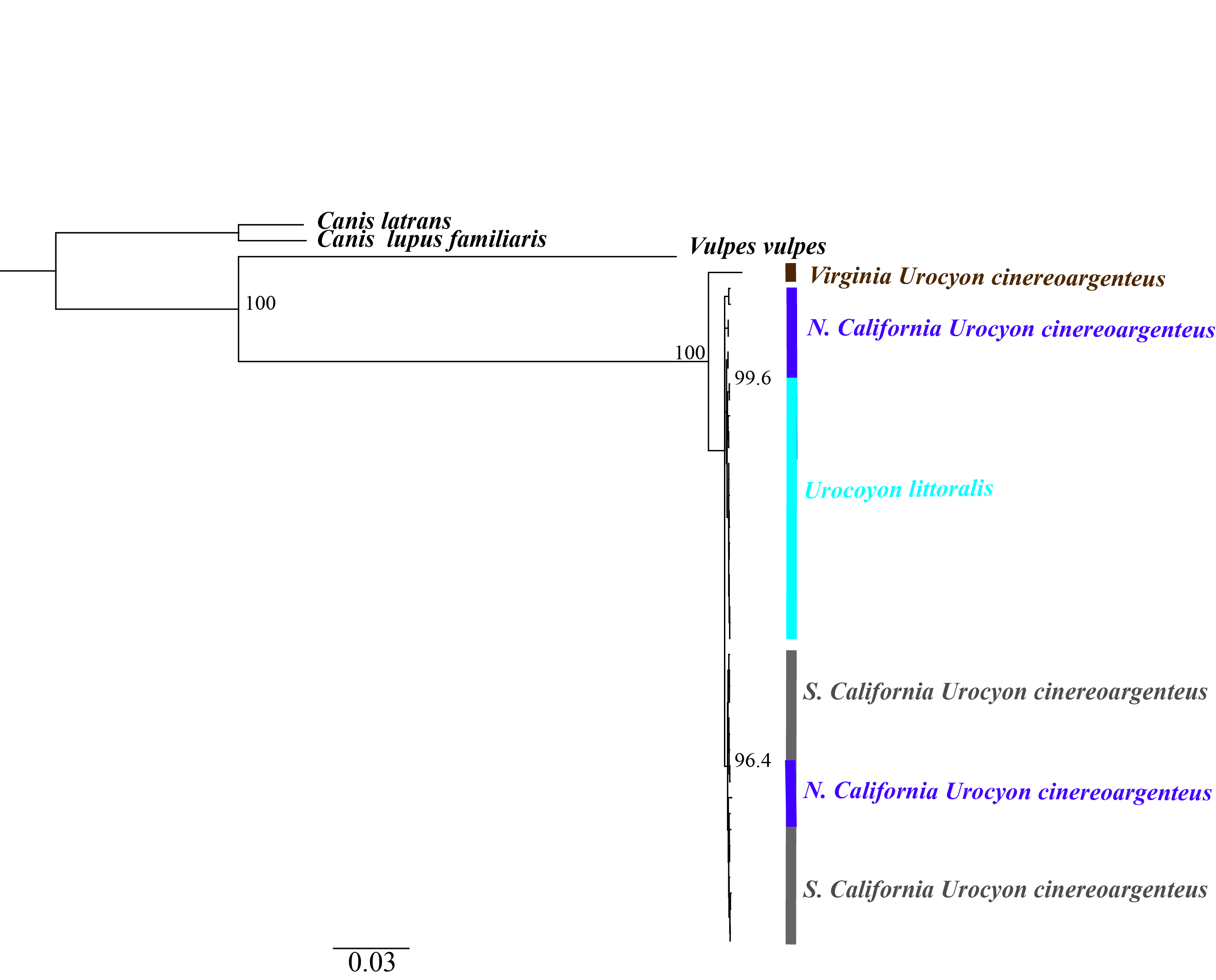

Supplement: S2 Fig — Rooted tree generated in Garli with 1000 bootstrap replicates. Key nodes are shown with bootstrap support. Nodes that are not labeled may also have strong support. Eastern gray fox is basal to the California clade and there is strong support for Clade A and Clade B (Fig. 3). (TIF) [file pone.0118240.s002.tif]

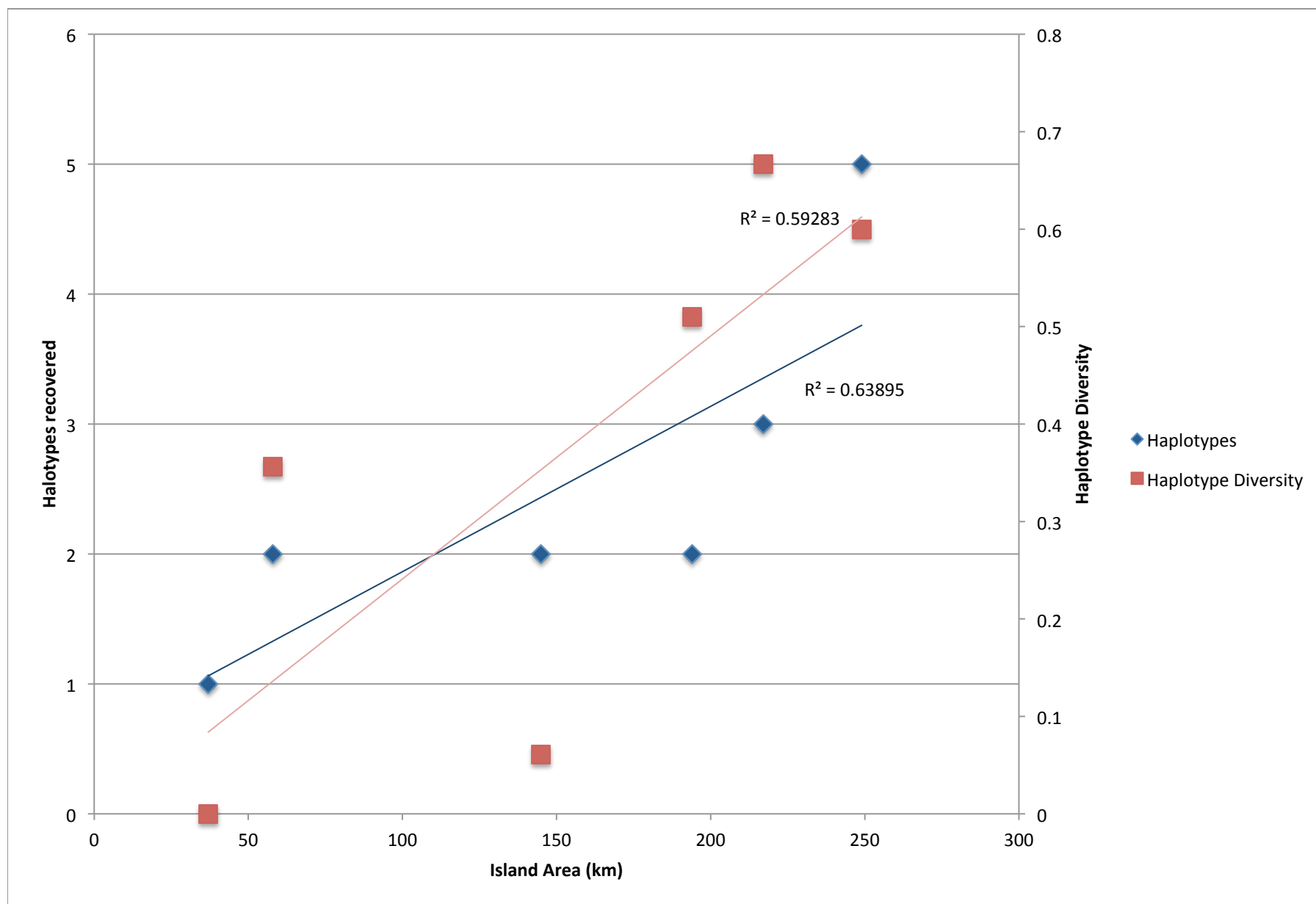

Supplement: S3 Fig — A positive correlation was identified between island area and the number of haplotypes recovered and haplotype diversity (Pearson’s r = 0.80 p-value = 0.03 and r = 0.77 p-value = 0.04, respectively). (PDF) [file pone.0118240.s003.pdf]
