# Supplementary material for: Mitochondrial Genomes Suggest Rapid Evolution of Dwarf California Channel Islands Foxes (Urocyon littoralis)
Source: PLoS One. 2015 Feb 25;10(2):e0118240. doi: 10.1371/journal.pone.0118240 (PMC4340941; doi:10.1371/journal.pone.0118240)
Supplement: S1 Table — (DOCX) [file pone.0118240.s005.docx]

Table S1: Sample and Coverage Information

| **Sample ID** | **Location``** | **UTM Easting** | **UTM Northing** | **Collection** | **Tissue type** | **Extraction Kit** | **Library Prep Type** | **Platform** | **Lane** | **Mean Read Depth** | **Read Depth**  **Std. Dev.** | **MtDNA Haplotype** |
| --- | --- | --- | --- | --- | --- | --- | --- | --- | --- | --- | --- | --- |
| MVZ-225021 | Heggenberger Parkway Golf Course, Alameda | 570630 | 4175095 | The Museum of Vertebrate Zoology at Berkeley | Tissue | Qiagen Blood and Tissue Kit | 454-Long Range PCR | 454 Jr. | Run 1 | 66.61 | 23.5163 | 1 |
| MVZ-225290 | jct Harbor Bay Parkway and Ron Cowan Parkway, Alameda | 568030 | 4175862 | The Museum of Vertebrate Zoology at Berkeley | Tissue | Qiagen Blood and Tissue Kit | 454-Long Range PCR | 454 Jr. | Run 1 | 64.32 | 29.2356 | 1 |
| MVZ-225670 | 22872 Walnut Blvd., Walnut Creek | 584480 | 4194730 | The Museum of Vertebrate Zoology at Berkeley | Tissue | Qiagen Blood and Tissue Kit | 454-Long Range PCR | 454 Jr. | Run 1 | 55.38 | 50.3052 | 3 |
| MVZ-218726 | Pinole Valley Rd. near Castro Ranch Rd., Pinole | 565082 | 4203607 | The Museum of Vertebrate Zoology at Berkeley | Tissue | Qiagen Blood and Tissue Kit | 454-Long Range PCR | 454 Jr. | Run 1 | 235.26 | 89.9407 | 4 |
| MVZ-225294 | Wldcat Canyon Road, just S Black Butte | 553173 | 4582121 | The Museum of Vertebrate Zoology at Berkeley | Tissue | Qiagen Blood and Tissue Kit | 454-Long Range PCR | 454 Jr. | Run 1 | 116.71 | 42.8173 | 5 |
| 9453C/120411 | Catalina Island | 368354 | 3694533 | Santa Barbara Museum of Natural History | Tongue | Qiagen Blood and Tissue Kit | 454-Long Range PCR | 454 Jr. | Run 1 | 117.33 | 47.3183 | 30 |
| 48611 | Catalina Island, East End | 375993 | 3690332 | Catalina Island Conservancy | Blood clot | Qiagen Blood and Tissue Kit | Nextera style- Long Range PCR | Illumina HiSeq | Lane 1 | 3427.89 | 2250.56 | 31 |
| 49148 | Catalina Island, East End | 367290 | 3696489 | Catalina Island Conservancy | Blood clot | Qiagen Blood and Tissue Kit | Nextera style- Long Range PCR | Illumina HiSeq | Lane 1 | 5042.92 | 2769.8 | 30 |
| 51828 | Catalina Island, West End | 359824 | 3702581 | Catalina Island Conservancy | Blood clot | Qiagen Blood and Tissue Kit | Nextera style- Long Range PCR | Illumina HiSeq | Lane 1 | 4212.8 | 1635.43 | 31 |
| 51F0D | Catalina Island, East End | 374297 | 3688024 | Catalina Island Conservancy | Blood clot | Qiagen Blood and Tissue Kit | Nextera style- Long Range PCR | Illumina HiSeq | Lane 1 | 4607.1 | 2889.04 | 31 |
| 52322 | Catalina Island, East End | 363233 | 3699211 | Catalina Island Conservancy | Blood clot | Qiagen Blood and Tissue Kit | Nextera style- Long Range PCR | Illumina HiSeq | Lane 1 | 4499.52 | 1926.41 | 30 |
| 53727 | Catalina Island, East End | 375339 | 3688471 | Catalina Island Conservancy | Blood clot | Qiagen Blood and Tissue Kit | Nextera style- Long Range PCR | Illumina HiSeq | Lane 1 | 5380.94 | 2332.61 | 31 |
| 53740 | Catalina Island, West End | 356996 | 3704475 | Catalina Island Conservancy | Blood clot | Qiagen Blood and Tissue Kit | Nextera style- Long Range PCR | Illumina HiSeq | Lane 1 | 7316.77 | 882.234 | 30 |
| 58834 | Catalina Island, West End | 358482 | 3702357 | Catalina Island Conservancy | Blood clot | Qiagen Blood and Tissue Kit | Nextera style- Long Range PCR | Illumina HiSeq | Lane 1 | 5678.15 | 2174.25 | 30 |
| 59414 | Catalina Island, East End | 367176 | 3694989 | Catalina Island Conservancy | Blood clot | Qiagen Blood and Tissue Kit | Nextera style- Long Range PCR | Illumina HiSeq | Lane 1 | 1595.85 | 1395.2 | 30 |
| 61367 | Catalina Island, West End | 358833 | 3701255 | Catalina Island Conservancy | Blood clot | Qiagen Blood and Tissue Kit | Nextera style- Long Range PCR | Illumina HiSeq | Lane 1 | 2011.7 | 1944.71 | 30 |
| 62669 | Catalina Island, West End | 358485 | 3702613 | Catalina Island Conservancy | Blood clot | Qiagen Blood and Tissue Kit | Nextera style- Long Range PCR | Illumina HiSeq | Lane 1 | 2345.64 | 1901.61 | 31 |
| 63330 | Catalina Island, West End | 359503 | 3702350 | Catalina Island Conservancy | Blood clot | Qiagen Blood and Tissue Kit | Nextera style- Long Range PCR | Illumina HiSeq | Lane 1 | 3601.2 | 1949.5 | 31 |
| 63380 | Catalina Island, West End | 359864 | 3702167 | Catalina Island Conservancy | Blood clot | Qiagen Blood and Tissue Kit | Nextera style- Long Range PCR | Illumina HiSeq | Lane 1 | 1307.89 | 1504.71 | 31 |
| 63425 | Catalina Island, East End | 377525 | 3689600 | Catalina Island Conservancy | Blood clot | Qiagen Blood and Tissue Kit | Nextera style- Long Range PCR | Illumina HiSeq | Lane 1 | 1716.22 | 1634.61 | 30 |
| 71075 | Catalina Island, East End | 363466 | 3696922 | Catalina Island Conservancy | Blood clot | Qiagen Blood and Tissue Kit | Nextera style- Long Range PCR | Illumina HiSeq | Lane 1 | 6800.18 | 1183.22 | 31 |
| 74743 | Catalina Island, West End | 354918 | 3703133 | Catalina Island Conservancy | Blood clot | Qiagen Blood and Tissue Kit | Nextera style- Long Range PCR | Illumina HiSeq | Lane 1 | 7861.92 | 507.962 | 30 |
| 78022 | Catalina Island, East End | 365222 | 3699331 | Catalina Island Conservancy | Blood clot | Qiagen Blood and Tissue Kit | Nextera style- Long Range PCR | Illumina HiSeq | Lane 1 | 2888.8 | 2008.79 | 30 |
| 80157 | Catalina Island, East End | 365968 | 3695742 | Catalina Island Conservancy | Blood clot | Qiagen Blood and Tissue Kit | Nextera style- Long Range PCR | Illumina HiSeq | Lane 1 | 6929.32 | 1132.73 | 30 |
| 85606 | Catalina Island, West End | 359643 | 3700359 | Catalina Island Conservancy | Blood clot | Qiagen Blood and Tissue Kit | Nextera style- Long Range PCR | Illumina HiSeq | Lane 1 | 69.0757 | 480.657 | 31 |
| 85661 | Catalina Island, East End | 371212 | 3692173 | Catalina Island Conservancy | Blood clot | Qiagen Blood and Tissue Kit | Nextera style- Long Range PCR | Illumina HiSeq | Lane 1 | 4733.57 | 2125.59 | 31 |
| 85871 | Catalina Island, West End | 353274 | 3703555 | Catalina Island Conservancy | Blood clot | Qiagen Blood and Tissue Kit | Nextera style- Long Range PCR | Illumina HiSeq | Lane 1 | 3248.91 | 2925.76 | 30 |
| 86740 | Catalina Island, West End | 356996 | 3704475 | Catalina Island Conservancy | Blood clot | Qiagen Blood and Tissue Kit | Nextera style- Long Range PCR | Illumina HiSeq | Lane 1 | 3879.08 | 3456.81 | 30 |
| 86765 | Catalina Island, East End | 374122 | 3690281 | Catalina Island Conservancy | Blood clot | Qiagen Blood and Tissue Kit | Nextera style- Long Range PCR | Illumina HiSeq | Lane 1 | 3758.87 | 1786.74 | 30 |
| 86784 | Catalina Island, East End | 361306 | 3701255 | Catalina Island Conservancy | Blood clot | Qiagen Blood and Tissue Kit | Nextera style- Long Range PCR | Illumina HiSeq | Lane 1 | 3039.88 | 1927.83 | 30 |
| 87067 | Catalina Island, West End | 359998 | 3702140 | Catalina Island Conservancy | Blood clot | Qiagen Blood and Tissue Kit | Nextera style- Long Range PCR | Illumina HiSeq | Lane 1 | 2376.5 | 1915.92 | 31 |
| 87682 | Catalina Island, East End | 370256 | 3694001 | Catalina Island Conservancy | Blood clot | Qiagen Blood and Tissue Kit | Nextera style- Long Range PCR | Illumina HiSeq | Lane 1 | 1084.94 | 1408.88 | 31 |
| 87785 | Catalina Island, West End | 355778 | 3704312 | Catalina Island Conservancy | Blood clot | Qiagen Blood and Tissue Kit | Nextera style- Long Range PCR | Illumina HiSeq | Lane 1 | 2852.75 | 1588.71 | 30 |
| 87970 | Catalina Island, East End | 368770 | 3696998 | Catalina Island Conservancy | Blood clot | Qiagen Blood and Tissue Kit | Nextera style- Long Range PCR | Illumina HiSeq | Lane 1 | 4685.53 | 1575.4 | 31 |
| 88196 | Catalina Island, West End | 359503 | 3702350 | Catalina Island Conservancy | Blood clot | Qiagen Blood and Tissue Kit | Nextera style- Long Range PCR | Illumina HiSeq | Lane 1 | 1634.84 | 1499.63 | 31 |
| 88351 | Catalina Island, East End | 376680 | 3687253 | Catalina Island Conservancy | Blood clot | Qiagen Blood and Tissue Kit | Nextera style- Long Range PCR | Illumina HiSeq | Lane 1 | 3017.61 | 1797.82 | 30 |
| 88406 | Catalina Island, West End | 359347 | 3702723 | Catalina Island Conservancy | Blood clot | Qiagen Blood and Tissue Kit | Nextera style- Long Range PCR | Illumina HiSeq | Lane 1 | 5928.68 | 1422.08 | 31 |
| 90186 | Catalina Island, West End | 356117 | 3704799 | Catalina Island Conservancy | Blood clot | Qiagen Blood and Tissue Kit | Nextera style- Long Range PCR | Illumina HiSeq | Lane 1 | 7875.41 | 456.5 | 30 |
| 90302 | Catalina Island, East End | 365411 | 3695162 | Catalina Island Conservancy | Blood clot | Qiagen Blood and Tissue Kit | Nextera style- Long Range PCR | Illumina HiSeq | Lane 1 | 5832.92 | 1665.35 | 31 |
| 92421 | Catalina Island, East End | 363147 | 3695845 | Catalina Island Conservancy | Blood clot | Qiagen Blood and Tissue Kit | Nextera style- Long Range PCR | Illumina HiSeq | Lane 1 | 3568.63 | 3855.16 | 31 |
| 94745 | Catalina Island, East End | 366675 | 3691623 | Catalina Island Conservancy | Blood clot | Qiagen Blood and Tissue Kit | Nextera style- Long Range PCR | Illumina HiSeq | Lane 1 | 7010.7 | 977.156 | 31 |
| 94867 | Catalina Island, West End | 353968 | 3704552 | Catalina Island Conservancy | Blood clot | Qiagen Blood and Tissue Kit | Nextera style- Long Range PCR | Illumina HiSeq | Lane 1 | 7822.25 | 621.734 | 30 |
| 95091 | Catalina Island, East End | 368953 | 3695210 | Catalina Island Conservancy | Blood clot | Qiagen Blood and Tissue Kit | Nextera style- Long Range PCR | Illumina HiSeq | Lane 1 | 1735.29 | 1230.84 | 31 |
| 97686 | Catalina Island, East End | 368773 | 3692605 | Catalina Island Conservancy | Blood clot | Qiagen Blood and Tissue Kit | Nextera style- Long Range PCR | Illumina HiSeq | Lane 1 | 7817.16 | 591.471 | 31 |
| UNK22 | Catalina Island | 372011 | 3693095 | Santa Barbara Museum of Natural History | Tongue | Qiagen Blood and Tissue Kit | 454-Long Range PCR | 454 Jr. | Run 1 | 140.52 | 48.2544 | 31 |
| UNK23 | Catalina Island | 368354 | 3694533 | Santa Barbara Museum of Natural History | Tongue | Qiagen Blood and Tissue Kit | 454-Long Range PCR | 454 Jr. | Run 1 | 154.89 | 50.228 | 31 |
| 985120029035544 | San Clemente Island | 350553 | 3654218 | Colorado State University, W. Chris Funk | Blood | Qiagen Blood- BioSprint | Nextera style-Capture | Illumina HiSeq | Lane 2 | 1120.83 | 390.427 | 33 |
| 4451290268 | San Clemente Island | 351891 | 3654581 | , Colorado State University, W. Chris Funk | Blood | Qiagen Blood- BioSprint | Nextera style-Capture | Illumina HiSeq | Lane 2 | 2523.52 | 947.248 | 33 |
| 985120027742591 | San Clemente Island | 357231.1979 | 3648401.225 | Colorado State University, W. Chris Funk | Blood | Qiagen Blood- BioSprint | Nextera style-Capture | Illumina HiSeq | Lane 2 | 201.066 | 64.2839 | 33 |
| 985120027742865 | San Clemente Island | 351171.1979 | 3654131.225 | Colorado State University, W. Chris Funk | Blood | Qiagen Blood- BioSprint | Nextera style-Capture | Illumina HiSeq | Lane 2 | 7881.54 | 388.872 | 33 |
| 985120028532610 | San Clemente Island | 356301.1979 | 3642851.225 | Colorado State University, W. Chris Funk | Blood | Qiagen Blood- BioSprint | Nextera style-Capture | Illumina HiSeq | Lane 2 | 6272.4 | 2055.79 | 33 |
| 985120028546728 | San Clemente Island | 351891 | 3654581 | Colorado State University, W. Chris Funk | Blood | Qiagen Blood- BioSprint | Nextera style-Capture | Illumina HiSeq | Lane 2 | 5006.84 | 1791.85 | 33 |
| 985120028551893 | San Clemente Island | 356077 | 3643519 | Colorado State University, W. Chris Funk | Blood | Qiagen Blood- BioSprint | Nextera style-Capture | Illumina HiSeq | Lane 2 | 1958.24 | 638.868 | 33 |
| 985120028845967 | San Clemente Island | 356541.1979 | 3644111.225 | Colorado State University, W. Chris Funk | Blood | Qiagen Blood- BioSprint | Nextera style-Capture | Illumina HiSeq | Lane 2 | 7770.71 | 790.693 | 33 |
| 985120028866749 | San Clemente Island | 359751 | 3644651 | Colorado State University, W. Chris Funk | Blood | Qiagen Blood- BioSprint | Nextera style-Capture | Illumina HiSeq | Lane 2 | 3561.29 | 1426.61 | 33 |
| 985120028892352 | San Clemente Island | 363861 | 3638951 | Colorado State University, W. Chris Funk | Blood | Qiagen Blood- BioSprint | Nextera style-Capture | Illumina HiSeq | Lane 2 | 6506.04 | 1634.1 | 33 |
| 985120028919325 | San Clemente Island | 350541 | 3654941 | Colorado State University, W. Chris Funk | Blood | Qiagen Blood- BioSprint | Nextera style-Capture | Illumina HiSeq | Lane 2 | 7886.36 | 356.804 | 33 |
| 985120029004811 | San Clemente Island | 356631 | 3648161 | Colorado State University, W. Chris Funk | Blood | Qiagen Blood- BioSprint | Nextera style-Capture | Illumina HiSeq | Lane 2 | 2046.23 | 985.227 | 33 |
| 985120029005330 | San Clemente Island | 355551.1979 | 3650171.225 | Colorado State University, W. Chris Funk | Blood | Qiagen Blood- BioSprint | Nextera style-Capture | Illumina HiSeq | Lane 2 | 7865.8 | 449.059 | 33 |
| 985120030856620 | San Clemente Island | 359571.1979 | 3639071.225 | Colorado State University, W. Chris Funk | Blood | Qiagen Blood- BioSprint | Nextera style-Capture | Illumina HiSeq | Lane 2 | 7885.4 | 385.475 | 32 |
| 985120030860616 | San Clemente Island | 355011.1979 | 3646481.225 | Colorado State University, W. Chris Funk | Blood | Qiagen Blood- BioSprint | Nextera style-Capture | Illumina HiSeq | Lane 2 | 3221.71 | 1419.21 | 33 |
| 985120030899238 | San Clemente Island | 362391.1979 | 3637511.225 | Colorado State University, W. Chris Funk | Blood | Qiagen Blood- BioSprint | Nextera style-Capture | Illumina HiSeq | Lane 2 | 85.8404 | 27.283 | 33 |
| 985120030920834 | San Clemente Island | 356751 | 3647351 | Colorado State University, W. Chris Funk | Blood | Qiagen Blood- BioSprint | Nextera style-Capture | Illumina HiSeq | Lane 2 | 7878.75 | 391.087 | 33 |
| 985120030923763 | San Clemente Island | 362031.1979 | 3640241.225 | Colorado State University, W. Chris Funk | Blood | Qiagen Blood- BioSprint | Nextera style-Capture | Illumina HiSeq | Lane 2 | 1174.54 | 392.522 | 33 |
| 985120031189688 | San Clemente Island | 356571.1979 | 3643601.225 | Colorado State University, W. Chris Funk | Blood | Qiagen Blood- BioSprint | Nextera style-Capture | Illumina HiSeq | Lane 2 | 4968.23 | 1904.79 | 33 |
| 985120031586905 | San Clemente Island | 356288.1258 | 3648385.698 | Colorado State University, W. Chris Funk | Blood | Qiagen Blood- BioSprint | Nextera style-Capture | Illumina HiSeq | Lane 2 | 7653.22 | 763.475 | 33 |
| 985120032123337 | San Clemente Island | 353001.1979 | 3649361.225 | Colorado State University, W. Chris Funk | Blood | Qiagen Blood- BioSprint | Nextera style-Capture | Illumina HiSeq | Lane 2 | 5362.98 | 1619.35 | 33 |
| 985120032205152 | San Clemente Island | 356631 | 3648161 | Colorado State University, W. Chris Funk | Blood | Qiagen Blood- BioSprint | Nextera style-Capture | Illumina HiSeq | Lane 2 | 7688.08 | 753.905 | 33 |
| 985120032253498 | San Clemente Island | 356391.1979 | 3645281.225 | Colorado State University, W. Chris Funk | Blood | Qiagen Blood- BioSprint | Nextera style-Capture | Illumina HiSeq | Lane 2 | 2298.45 | 833.817 | 33 |
| 985120032342258 | San Clemente Island | 352881.1979 | 3650681.225 | Colorado State University, W. Chris Funk | Blood | Qiagen Blood- BioSprint | Nextera style-Capture | Illumina HiSeq | Lane 2 | 1592.07 | 555.123 | 33 |
| 985120032346926 | San Clemente Island | 355131.1979 | 3650741.225 | Colorado State University, W. Chris Funk | Blood | Qiagen Blood- BioSprint | Nextera style-Capture | Illumina HiSeq | Lane 2 | 5817.54 | 1903.74 | 33 |
| 985120032372226 | San Clemente Island | 352344.2787 | 3653793.885 | Colorado State University, W. Chris Funk | Blood | Qiagen Blood- BioSprint | Nextera style-Capture | Illumina HiSeq | Lane 2 | 7804.06 | 586.673 | 33 |
| 985120032504581 | San Clemente Island | 352761.1979 | 3652001.225 | Colorado State University, W. Chris Funk | Blood | Qiagen Blood- BioSprint | Nextera style-Capture | Illumina HiSeq | Lane 2 | 6225.38 | 1611.52 | 33 |
| 985120032532213 | San Clemente Island | 360771.1979 | 3641381.225 | Colorado State University, W. Chris Funk | Blood | Qiagen Blood- BioSprint | Nextera style-Capture | Illumina HiSeq | Lane 2 | 33.6857 | 16.0528 | 33 |
| 430F762301 | San Clemente Island | 358041.1979 | 3642311.225 | Colorado State University, W. Chris Funk | Blood | Qiagen Blood- BioSprint | Nextera style-Capture | Illumina HiSeq | Lane 2 | 2639.16 | 717.947 | 33 |
| 454C113F32 | San Clemente Island | 351891 | 3654581 | Colorado State University, W. Chris Funk | Blood | Qiagen Blood- BioSprint | Nextera style-Capture | Illumina HiSeq | Lane 2 | 7792.93 | 602.367 | 33 |
| 4A44416734 | San Clemente Island | 357921.1979 | 3646481.225 | Colorado State University, W. Chris Funk | Blood | Qiagen Blood- BioSprint | Nextera style-Capture | Illumina HiSeq | Lane 2 | 7860.43 | 433.234 | 33 |
| 4A5867027B | San Clemente Island | 359751 | 3644651 | Colorado State University, W. Chris Funk | Blood | Qiagen Blood- BioSprint | Nextera style-Capture | Illumina HiSeq | Lane 2 | 3839.99 | 127.15 | 33 |
| 4658713E74 | San Clemente Island | 360381.1979 | 3636941.225 | Colorado State University, W. Chris Funk | Blood | Qiagen Blood- BioSprint | Nextera style-Capture | Illumina HiSeq | Lane 2 | 985.542 | 325.384 | 33 |
| MVZ-225303 | Carmel Valley Road, just south of Carmel Valley Village | 616886 | 4034993 | The Museum of Vertebrate Zoology at Berkeley | Tissue | Qiagen Blood and Tissue Kit | 454-Long Range PCR | 454 Jr. | Run 1 | 143.94 | 80.0184 | 6 |
| 01PANT-20090525 |  | 11/427690 | 3725840 | Colorado State University, W. Chris Funk | Blood | Qiagen Blood- BioSprint | Nextera style-Capture | Illumina HiSeq | Lane 2 | 2068.05 | 607.405 | 12 |
| 01PEAN-20090302 |  | 11/427690 | 3725840 | Colorado State University, W. Chris Funk | Blood | Qiagen Blood- BioSprint | Nextera style-Capture | Illumina HiSeq | Lane 2 | 1397.43 | 449.111 | 19 |
| 01PCLE-20090203 |  | 11/594494 | 3732687 | Colorado State University, W. Chris Funk | Blood | Qiagen Blood- BioSprint | Nextera style-Capture | Illumina HiSeq | Lane 2 | 2717.33 | 958.824 | 12 |
| 01PROB-20090109 |  | 11/594494 | 3732687 | Colorado State University, W. Chris Funk | Blood | Qiagen Blood- BioSprint | Nextera style-Capture | Illumina HiSeq | Lane 2 | 1479.14 | 452.484 | 16 |
| MVZ-225009 | Fair Oaks region, Sacramento | 650540 | 4279848 | The Museum of Vertebrate Zoology at Berkeley | Tissue | Qiagen Blood and Tissue Kit | 454-Long Range PCR | 454 Jr. | Run 1 | 106.81 | 56.9379 | 7 |
| F08 |  | 11/521481 | 3653528 | Colorado State University, W. Chris Funk | Tongue | Qiagen Blood and Tissue Kit | Nextera style-Capture | Illumina HiSeq | Lane 2 | 6989 | 1066.72 | 16 |
| F11 |  | 11/521481 | 3653528 | Colorado State University, W. Chris Funk | Tongue | Qiagen Blood and Tissue Kit | Nextera style-Capture | Illumina HiSeq | Lane 2 | 7306.63 | 1310.01 | 10 |
| PWC-5206 | East End of Santa Ynez Valley, Hwy 154 adj overlook just W of Cold Spring Bridge | 239182 | 3824612 | Santa Barbara Museum of Natural History | Muscle | Qiagen Blood and Tissue Kit | Nextera style-Capture | Illumina HiSeq | Lane 2 | 7891.7 | 207.384 | 18 |
| PWC-5213 | Hollister Ranch, access rd at jct Parcels 125 X 126 | 753372 | 3817693 | Santa Barbara Museum of Natural History | Muscle | Qiagen Blood and Tissue Kit | Nextera style-Capture | Illumina HiSeq | Lane 2 | 7765.68 | 694.064 | 21 |
| CHIS30121 | San Miguel Island | 745212 | 3769204 | American Museum of Natural History | Blood | Qiagen Blood and Tissue Kit | Nextera style- Long Range PCR | Illumina HiSeq | Lane 1 | 7782.49 | 722.372 | 29 |
| CHIS30361 | San Miguel Island | 744309 | 3769463 | American Museum of Natural History | Blood | Qiagen Blood and Tissue Kit | Nextera style- Long Range PCR | Illumina HiSeq | Lane 1 | 7657.11 | 864.538 | 29 |
| CHIS30415 | San Miguel Island | 744189 | 3769552 | American Museum of Natural History | Blood | Qiagen Blood and Tissue Kit | Nextera style- Long Range PCR | Illumina HiSeq | Lane 1 | 7774.29 | 724.807 | 29 |
| CHIS30091 | San Miguel Island | 744728 | 3769504 | American Museum of Natural History | Blood | Qiagen Blood and Tissue Kit | Nextera style- Long Range PCR | Illumina HiSeq | Lane 1 | 7735.81 | 837.032 | 29 |
| CHIS30061 | San Miguel Island | 742909 | 3770227 | American Museum of Natural History | Blood | Qiagen Blood and Tissue Kit | Nextera style- Long Range PCR | Illumina HiSeq | Lane 1 | 4804.53 | 1681.08 | 29 |
| CHIS30399 | San Miguel Island | 745063 | 3769184 | American Museum of Natural History | Blood | Qiagen Blood and Tissue Kit | Nextera style- Long Range PCR | Illumina HiSeq | Lane 1 | 6535.43 | 1100.37 | 29 |
| CHIS30373 | San Miguel Island | 744641 | 3769582 | American Museum of Natural History | Blood | Qiagen Blood and Tissue Kit | Nextera style- Long Range PCR | Illumina HiSeq | Lane 1 | 7844 | 570.698 | 29 |
| CHIS30459 | San Miguel Island | 744857 | 3769643 | American Museum of Natural History | Blood | Qiagen Blood and Tissue Kit | Nextera style- Long Range PCR | Illumina HiSeq | Lane 1 | 7768.76 | 806.542 | 29 |
| CHIS30388 | San Miguel Island | 744600 | 3769382 | American Museum of Natural History | Blood | Qiagen Blood and Tissue Kit | Nextera style- Long Range PCR | Illumina HiSeq | Lane 1 | 5350.13 | 1301.11 | 29 |
| CHIS30118 | San Miguel Island | 744413 | 3769859 | American Museum of Natural History | Blood | Qiagen Blood and Tissue Kit | Nextera style- Long Range PCR | Illumina HiSeq | Lane 1 | 7273.61 | 1067.55 | 29 |
| CHIS30432 | San Miguel Island | 744330 | 3769867 | American Museum of Natural History | Blood | Qiagen Blood and Tissue Kit | 454-Long Range PCR | 454 Jr. | Run 1 | 282.14 | 151.513 | 29 |
| CHIS30092 | San Miguel Island | 744365 | 3768890 | American Museum of Natural History | Blood | Qiagen Blood and Tissue Kit | Nextera style- Long Range PCR | Illumina HiSeq | Lane 1 | 7867.1 | 487.685 | 29 |
| CHIS30120 | San Miguel Island | 745348 | 3769299 | American Museum of Natural History | Blood | Qiagen Blood and Tissue Kit | Nextera style- Long Range PCR | Illumina HiSeq | Lane 1 | 7739.17 | 822.661 | 29 |
| CHIS30417 | San Miguel Island | 745242 | 3769952 | American Museum of Natural History | Blood | Qiagen Blood and Tissue Kit | Nextera style- Long Range PCR | Illumina HiSeq | Lane 1 | 6771.27 | 1280.3 | 29 |
| CHIS30413 | San Miguel Island | 745202 | 3769515 | American Museum of Natural History | Blood | Qiagen Blood and Tissue Kit | Nextera style- Long Range PCR | Illumina HiSeq | Lane 1 | 5852.29 | 1388.28 | 29 |
| CHIS 30358 | San Miguel Island | 746625 | 3768017 | Colorado State University, W. Chris Funk | Blood clot | Qiagen Blood- BioSprint | Nextera style-Capture | Illumina HiSeq | Lane 2 | 3050.61 | 942.643 | 29 |
| CHIS 30362 | San Miguel Island | 743073 | 3768390 | Colorado State University, W. Chris Funk | Blood clot | Qiagen Blood- BioSprint | Nextera style-Capture | Illumina HiSeq | Lane 2 | 74.5681 | 24.4785 | 29 |
| CHIS 30363 | San Miguel Island | 743073 | 3768390 | Colorado State University, W. Chris Funk | Blood clot | Qiagen Blood- BioSprint | Nextera style-Capture | Illumina HiSeq | Lane 2 | 132.425 | 42.5343 | 29 |
| CHIS 30385 | San Miguel Island | 743073 | 3768390 | Colorado State University, W. Chris Funk | Blood clot | Qiagen Blood- BioSprint | Nextera style-Capture | Illumina HiSeq | Lane 2 | 1332.06 | 506.412 | 29 |
| CHIS 30392 | San Miguel Island | 746625 | 3768017 | Colorado State University, W. Chris Funk | Blood clot | Qiagen Blood- BioSprint | Nextera style-Capture | Illumina HiSeq | Lane 2 | 353.984 | 111.87 | 29 |
| CHIS 30424 | San Miguel Island | 746625 | 3768017 | Colorado State University, W. Chris Funk | Blood clot | Qiagen Blood- BioSprint | Nextera style-Capture | Illumina HiSeq | Lane 2 | 91.8899 | 32.4108 | 29 |
| CHIS 30441 | San Miguel Island | 746625 | 3768017 | Colorado State University, W. Chris Funk | Blood clot | Qiagen Blood- BioSprint | Nextera style-Capture | Illumina HiSeq | Lane 2 | 1957.38 | 592.496 | 29 |
| CHIS 30455 | San Miguel Island | 746625 | 3768017 | Colorado State University, W. Chris Funk | Blood clot | Qiagen Blood- BioSprint | Nextera style-Capture | Illumina HiSeq | Lane 2 | 122.705 | 39.5651 | 29 |
| F118 | Santa Cruz Island | 251576 | 3764658 | Colorado State University, W. Chris Funk | Blood clot | Qiagen Blood- BioSprint | Nextera style-Capture | Illumina HiSeq | Lane 2 | 1477.41 | 394.938 | 23 |
| F128 | Santa Cruz Island | 263238 | 3768496 | The Nature Conservancy | Blood | Qiagen Blood and Tissue Kit | Nextera style-Capture | Illumina HiSeq | Lane 2 | 7636.55 | 836.653 | 23 |
| F248 | Santa Cruz Island | 260451 | 3766432 | The Nature Conservancy | Blood | Qiagen Blood and Tissue Kit | Nextera style-Capture | Illumina HiSeq | Lane 2 | 7550.51 | 820.605 | 23 |
| F333 | Santa Cruz Island | 249282 | 3765621 | The Nature Conservancy | Blood | Qiagen Blood and Tissue Kit | Nextera style-Capture | Illumina HiSeq | Lane 2 | 7891.53 | 255.338 | 23 |
| F345 | Santa Cruz Island | 241344 | 3768036 | The Nature Conservancy | Blood | Qiagen Blood and Tissue Kit | Nextera style-Capture | Illumina HiSeq | Lane 2 | 7502.37 | 881.411 | 31 |
| F351 | Santa Cruz Island | 254362 | 3766209 | The Nature Conservancy | Blood | Qiagen Blood and Tissue Kit | Nextera style-Capture | Illumina HiSeq | Lane 2 | 4510.24 | 1127.87 | 24 |
| F400 | Santa Cruz Island | 238911 | 3765933 | The Nature Conservancy | Blood | Qiagen Blood and Tissue Kit | Nextera style-Capture | Illumina HiSeq | Lane 2 | 7696.32 | 817.685 | 31 |
| F419 | Santa Cruz Island | 263789 | 3768748 | The Nature Conservancy | Blood | Qiagen Blood and Tissue Kit | Nextera style-Capture | Illumina HiSeq | Lane 2 | 7625.03 | 827.591 | 23 |
| F435 | Santa Cruz Island | 252769 | 3765528 | The Nature Conservancy | Blood | Qiagen Blood and Tissue Kit | Nextera style-Capture | Illumina HiSeq | Lane 2 | 7751.79 | 625.22 | 23 |
| F438 | Santa Cruz Island | 244434 | 3763840 | The Nature Conservancy | Blood | Qiagen Blood and Tissue Kit | Nextera style-Capture | Illumina HiSeq | Lane 2 | 6926.05 | 1234.54 | 25 |
| F441 | Santa Cruz Island | 249506 | 3765004 | The Nature Conservancy | Blood | Qiagen Blood and Tissue Kit | Nextera style-Capture | Illumina HiSeq | Lane 2 | 113.244 | 67.5294 | 23 |
| F455 | Santa Cruz Island | 234103 | 3770620 | The Nature Conservancy | Blood | Qiagen Blood and Tissue Kit | Nextera style-Capture | Illumina HiSeq | Lane 2 | 5642.3 | 1475.64 | 25 |
| F458 | Santa Cruz Island | 235893 | 3766001 | The Nature Conservancy | Blood | Qiagen Blood and Tissue Kit | Nextera style-Capture | Illumina HiSeq | Lane 2 | 7875.76 | 254.521 | 25 |
| M226 | Santa Cruz Island | 261430 | 3770226 | The Nature Conservancy | Blood | Qiagen Blood and Tissue Kit | Nextera style-Capture | Illumina HiSeq | Lane 2 | 7632.98 | 983.112 | 23 |
| M245 | Santa Cruz Island | 241716 | 3767298 | The Nature Conservancy | Blood | Qiagen Blood and Tissue Kit | Nextera style-Capture | Illumina HiSeq | Lane 2 | 5990.91 | 1484.01 | 23 |
| M246 | Santa Cruz Island | 244230 | 3765828 | The Nature Conservancy | Blood | Qiagen Blood and Tissue Kit | Nextera style-Capture | Illumina HiSeq | Lane 2 | 3783.87 | 1037.55 | 23 |
| M292 | Santa Cruz Island | 244067 | 3763681 | The Nature Conservancy | Blood | Qiagen Blood and Tissue Kit | Nextera style-Capture | Illumina HiSeq | Lane 2 | 5539.54 | 1538.43 | 23 |
| M315 | Santa Cruz Island | 252932 | 3766690 | The Nature Conservancy | Blood | Qiagen Blood and Tissue Kit | Nextera style-Capture | Illumina HiSeq | Lane 2 | 7832.71 | 476.22 | 23 |
| M364 | Santa Cruz Island | 245391 | 3761692 | The Nature Conservancy | Blood | Qiagen Blood and Tissue Kit | Nextera style-Capture | Illumina HiSeq | Lane 2 | 3085.73 | 961.816 | 24 |
| M409 | Santa Cruz Island | 231917 | 3773716 | The Nature Conservancy | Blood | Qiagen Blood and Tissue Kit | Nextera style-Capture | Illumina HiSeq | Lane 2 | 7838.29 | 504.867 | 25 |
| M434 | Santa Cruz Island | 263641 | 3766831 | The Nature Conservancy | Blood | Qiagen Blood and Tissue Kit | Nextera style-Capture | Illumina HiSeq | Lane 2 | 2756.84 | 891.341 | 23 |
| M444 | Santa Cruz Island | 239914 | 3765981 | The Nature Conservancy | Blood | Qiagen Blood and Tissue Kit | Nextera style-Capture | Illumina HiSeq | Lane 2 | 2952.37 | 1011.89 | 31 |
| M481 | Santa Cruz Island | 260451 | 3766432 | The Nature Conservancy | Blood | Qiagen Blood and Tissue Kit | Nextera style-Capture | Illumina HiSeq | Lane 2 | 7448.16 | 952.397 | 23 |
| M495 | Santa Cruz Island | 254680 | 3766524 | The Nature Conservancy | Blood | Qiagen Blood and Tissue Kit | Nextera style-Capture | Illumina HiSeq | Lane 2 | 7898.22 | 204.217 | 23 |
| M496 | Santa Cruz Island | 243700 | 3763522 | The Nature Conservancy | Blood | Qiagen Blood and Tissue Kit | Nextera style-Capture | Illumina HiSeq | Lane 2 | 4175.38 | 1095.58 | 22 |
| M512 | Santa Cruz Island | 264045 | 3766843 | The Nature Conservancy | Blood | Qiagen Blood and Tissue Kit | Nextera style-Capture | Illumina HiSeq | Lane 2 | 3890.18 | 1107.42 | 23 |
| M529 | Santa Cruz Island | 250500 | 3764612 | The Nature Conservancy | Blood | Qiagen Blood and Tissue Kit | Nextera style-Capture | Illumina HiSeq | Lane 2 | 7630.14 | 843.027 | 23 |
| M537 | Santa Cruz Island | 243837 | 3765905 | The Nature Conservancy | Blood | Qiagen Blood and Tissue Kit | Nextera style-Capture | Illumina HiSeq | Lane 2 | 7771.23 | 507.818 | 23 |
| F211 | Santa Cruz Island | 236129 | 3765611 | Colorado State University, W. Chris Funk | Blood clot | Qiagen Blood- BioSprint | Nextera style-Capture | Illumina HiSeq | Lane 2 | 1457.32 | 417.262 | 25 |
| F275 | Santa Cruz Island | 236646 | 3763944 | Colorado State University, W. Chris Funk | Blood clot | Qiagen Blood- BioSprint | Nextera style-Capture | Illumina HiSeq | Lane 2 | 1072.49 | 322.489 | 25 |
| F414 | Santa Cruz Island | 248789 | 3765253 | Colorado State University, W. Chris Funk | Blood clot | Qiagen Blood- BioSprint | Nextera style-Capture | Illumina HiSeq | Lane 2 | 4701.27 | 1411.9 | 23 |
| F446 | Santa Cruz Island | 236837 | 3763881 | Colorado State University, W. Chris Funk | Blood clot | Qiagen Blood- BioSprint | Nextera style-Capture | Illumina HiSeq | Lane 2 | 7371.47 | 912.294 | 31 |
| M178 | Santa Cruz Island | 236070 | 3766224 | Colorado State University, W. Chris Funk | Blood clot | Qiagen Blood- BioSprint | Nextera style-Capture | Illumina HiSeq | Lane 2 | 4195.68 | 1185.89 | 31 |
| M418 | Santa Cruz Island | 252211 | 3765098 | Colorado State University, W. Chris Funk | Blood clot | Qiagen Blood- BioSprint | Nextera style-Capture | Illumina HiSeq | Lane 2 | 207.436 | 101.071 | 23 |
| M425 | Santa Cruz Island | 236712 | 3763501 | Colorado State University, W. Chris Funk | Blood clot | Qiagen Blood- BioSprint | Nextera style-Capture | Illumina HiSeq | Lane 2 | 1656.9 | 486.808 | 31 |
| M429 | Santa Cruz Island | 235893 | 3766001 | Colorado State University, W. Chris Funk | Blood clot | Qiagen Blood- BioSprint | Nextera style-Capture | Illumina HiSeq | Lane 2 | 811.683 | 236.484 | 25 |
| M459 | Santa Cruz Island | 252251 | 3764842 | Colorado State University, W. Chris Funk | Blood clot | Qiagen Blood- BioSprint | Nextera style-Capture | Illumina HiSeq | Lane 2 | 702.687 | 198.842 | 23 |
| M465 | Santa Cruz Island | 252083 | 3764979 | Colorado State University, W. Chris Funk | Blood clot | Qiagen Blood- BioSprint | Nextera style-Capture | Illumina HiSeq | Lane 2 | 603.734 | 175.212 | 23 |
| M497 | Santa Cruz Island | 248984 | 3765047 | Colorado State University, W. Chris Funk | Blood clot | Qiagen Blood- BioSprint | Nextera style-Capture | Illumina HiSeq | Lane 2 | 1728.56 | 578.92 | 23 |
| M511 | Santa Cruz Island | 236587 | 3763122 | Colorado State University, W. Chris Funk | Blood clot | Qiagen Blood- BioSprint | Nextera style-Capture | Illumina HiSeq | Lane 2 | 371.381 | 133.89 | 25 |
| M561 | Santa Cruz Island | 248783 | 3764682 | Colorado State University, W. Chris Funk | Blood clot | Qiagen Blood- BioSprint | Nextera style-Capture | Illumina HiSeq | Lane 2 | 1809.74 | 525.887 | 23 |
| M562 | Santa Cruz Island | 248957 | 3764246 | Colorado State University, W. Chris Funk | Blood clot | Qiagen Blood- BioSprint | Nextera style-Capture | Illumina HiSeq | Lane 2 | 2786.65 | 878.635 | 23 |
| CHIS 30240 | Santa Rosa Island | 759981 | 3761422 | Colorado State University, W. Chris Funk | Blood clot | Qiagen Blood- BioSprint | Nextera style-Capture | Illumina HiSeq | Lane 2 | 6512.9 | 1391.47 | 28 |
| CHIS 30212 | Santa Rosa Island | 767592 | 3760504 | Colorado State University, W. Chris Funk | Blood clot | Qiagen Blood- BioSprint | Nextera style-Capture | Illumina HiSeq | Lane 2 | 4349.08 | 1281.73 | 27 |
| CHIS 30244 | Santa Rosa Island | 767592 | 3760504 | Colorado State University, W. Chris Funk | Blood clot | Qiagen Blood- BioSprint | Nextera style-Capture | Illumina HiSeq | Lane 2 | 1989.24 | 596.175 | 27 |
| CHIS 30255 | Santa Rosa Island | 772826 | 3761070 | Colorado State University, W. Chris Funk | Blood clot | Qiagen Blood- BioSprint | Nextera style-Capture | Illumina HiSeq | Lane 2 | 754.097 | 227.353 | 26 |
| CHIS 30298 | Santa Rosa Island | 772611 | 3761871 | Colorado State University, W. Chris Funk | Blood clot | Qiagen Blood- BioSprint | Nextera style-Capture | Illumina HiSeq | Lane 2 | 225.529 | 106.315 | 26 |
| CHIS 30328 | Santa Rosa Island | 759981 | 3761422 | Colorado State University, W. Chris Funk | Blood clot | Qiagen Blood- BioSprint | Nextera style-Capture | Illumina HiSeq | Lane 2 | 2317.86 | 695.888 | 27 |
| CHIS 30335 | Santa Rosa Island | 772826 | 3761070 | Colorado State University, W. Chris Funk | Blood clot | Qiagen Blood- BioSprint | Nextera style-Capture | Illumina HiSeq | Lane 2 | 330.34 | 103.171 | 26 |
| CHIS 30343 | Santa Rosa Island | 772611 | 3761871 | Colorado State University, W. Chris Funk | Blood clot | Qiagen Blood- BioSprint | Nextera style-Capture | Illumina HiSeq | Lane 2 | 222.988 | 70.744 | 26 |
| CHIS 30347 | Santa Rosa Island | 767592 | 3760504 | Colorado State University, W. Chris Funk | Blood clot | Qiagen Blood- BioSprint | Nextera style-Capture | Illumina HiSeq | Lane 2 | 250.266 | 79.5858 | 27 |
| PWC-5219 | Santa Ynez Valley, Brinkerhoff Rd, 1.5 mi N of Roblar Rd. | 770877 | 3840812 | Santa Barbara Museum of Natural History | Muscle | Qiagen Blood and Tissue Kit | Nextera style-Capture | Illumina HiSeq | Lane 2 | 2591.42 | 831.623 | 14 |
| PWC-5215 | Santa Ynez Valley, Hwy 154 | 11/240028.78 | 3823888.76 | Santa Barbara Museum of Natural History | Muscle | Qiagen Blood and Tissue Kit | Nextera style-Capture | Illumina HiSeq | Lane 2 | 7804.13 | 619.908 | 17 |
| PWC-5218 | Santa Ynez Valley, Hwy 154 0.3 mi W jct Paradise Rd. | 11/237118 | 3825792 | Santa Barbara Museum of Natural History | Muscle | Qiagen Blood and Tissue Kit | Nextera style-Capture | Illumina HiSeq | Lane 2 | 594.53 | 224.545 | 20 |
| PWC-5220 | Santa Ynez Valley, Hwy 154, ~ 2 mi E of Cachuma Reservoir | 11/239296 | 3824507 | Santa Barbara Museum of Natural History | Muscle | Qiagen Blood and Tissue Kit | Nextera style-Capture | Illumina HiSeq | Lane 2 | 7539.72 | 954.037 | 13 |
| PWC-5214 | Santa Ynez Valley, Hwy 154, ~1.5 miles W Jct entrance to Cachuma county campground | 11/225623 | 3830835 | Santa Barbara Museum of Natural History | Muscle | Qiagen Blood and Tissue Kit | Nextera style-Capture | Illumina HiSeq | Lane 2 | 7860.58 | 340.388 | 11 |
| MVZ-206290 | Hwy. 44, 0.25 mi W of Lassen Park Rd. | 620249 | 4488911 | The Museum of Vertebrate Zoology at Berkeley | Tissue | Qiagen Blood and Tissue Kit | 454-Long Range PCR | 454 Jr. | Run 1 | 171.66 | 51.659 | 5 |
| MVZ-218693 | 15 Corte Dorado, Benicia | 575094 | 4212822 | The Museum of Vertebrate Zoology at Berkeley | Tissue | Qiagen Blood and Tissue Kit | 454-Long Range PCR | 454 Jr. | Run 1 | 200.41 | 91.68 | 8 |
| MVZ-225297 | Hwy 36 at Canyon View Road | 604970 | 4466965 | The Museum of Vertebrate Zoology at Berkeley | Tissue | Qiagen Blood and Tissue Kit | 454-Long Range PCR | 454 Jr. | Run 1 | 117.85 | 39.7729 | 2 |
| MVZ-225296 | Hwy 36 at Morgan Summit | 624390 | 4469038 | The Museum of Vertebrate Zoology at Berkeley | Tissue | Qiagen Blood and Tissue Kit | 454-Long Range PCR | 454 Jr. | Run 1 | 181.17 | 75.2111 | 9 |
| PWC-5217 | Hwy 33 near Foster Park | 11/287643 | 3804254 | Santa Barbara Museum of Natural History | Tissue | Qiagen Blood and Tissue Kit | Nextera style-Capture | Illumina HiSeq | Lane 2 | 3954.62 | 1655.74 | 15 |
| 4A590A0977 | San Nicolas Island | 261667.0029 | 3683448.004 | Santa Barbara Museum of Natural History | Tongue | Qiagen Blood and Tissue Kit | 454-Long Range PCR | 454 Jr. | Run 1 | 532.88 | 189.258 | 35 |
| 4658582C6A | San Nicolas Island | 261667.0029 | 3683448.004 | Colorado State University, W. Chris Funk | Blood clot | Qiagen Blood- BioSprint | Nextera style-Capture | Illumina HiSeq | Lane 2 | 2891.15 | 904.681 | 35 |
| 47237B1B0B | San Nicolas Island | 268378.9973 | 3680792.008 | Colorado State University, W. Chris Funk | Blood clot | Qiagen Blood- BioSprint | Nextera style-Capture | Illumina HiSeq | Lane 2 | 1213.58 | 340.127 | 35 |
| 472C575E42 | San Nicolas Island | 268378.9973 | 3680792.008 | Colorado State University, W. Chris Funk | Blood clot | Qiagen Blood- BioSprint | Nextera style-Capture | Illumina HiSeq | Lane 2 | 733.362 | 264.141 | 34 |
| 4947314B0B | San Nicolas Island | 268378.9973 | 3680792.008 | Colorado State University, W. Chris Funk | Blood clot | Qiagen Blood- BioSprint | Nextera style-Capture | Illumina HiSeq | Lane 2 | 2140.64 | 725.618 | 35 |
| 49493E5D52 | San Nicolas Island | 261667.0029 | 3683448.004 | Colorado State University, W. Chris Funk | Blood clot | Qiagen Blood- BioSprint | Nextera style-Capture | Illumina HiSeq | Lane 2 | 968.068 | 320.046 | 35 |
| 4949A3D4D4C | San Nicolas Island | 261667.0029 | 3683448.004 | Colorado State University, W. Chris Funk | Blood clot | Qiagen Blood- BioSprint | Nextera style-Capture | Illumina HiSeq | Lane 2 | 663.243 | 204.88 | 35 |
| 4A5A0A3248 | San Nicolas Island | 268378.9973 | 3680792.008 | Colorado State University, W. Chris Funk | Blood clot | Qiagen Blood- BioSprint | Nextera style-Capture | Illumina HiSeq | Lane 2 | 1110.42 | 316.799 | 35 |
| 4B03640614 | San Nicolas Island | 268378.9973 | 3680792.008 | Colorado State University, W. Chris Funk | Blood clot | Qiagen Blood- BioSprint | Nextera style-Capture | Illumina HiSeq | Lane 2 | 1471.05 | 434.788 | 35 |
| 4B04785B14 | San Nicolas Island | 261667.0029 | 3683448.004 | Colorado State University, W. Chris Funk | Blood clot | Qiagen Blood- BioSprint | Nextera style-Capture | Illumina HiSeq | Lane 2 | 3223.38 | 1048.15 | 35 |
| CB17-1B Virginia | Woodstock, Virginia |  |  | CCEG Frozen Tissue Collection, Smithsonian Conservation Biology Institute |  |  | 454-Long Range PCR | 454 Jr. | Run 1 | 52.86 | 50.3052 | 36 |
