# Supplementary material for: Mitochondrial Genomes Suggest Rapid Evolution of Dwarf California Channel Islands Foxes (Urocyon littoralis)
Source: PLoS One. 2015 Feb 25;10(2):e0118240. doi: 10.1371/journal.pone.0118240 (PMC4340941; doi:10.1371/journal.pone.0118240)
Supplement: S2 Table — (DOCX) [file pone.0118240.s006.docx]

**Table S2. Distribution of polymorphisms in island and gray fox mitogenomes**

|  | **Property** | **Noncoding** | **Coding** | | | | **Total** |
| --- | --- | --- | --- | --- | --- | --- | --- |
|  |  | **D-loop** | **CytB** | **rRNA** | **tRNA** | **All Coding Genes** |  |
|  | Length (bp) | 992 | 1140 | 2547 | 1513 | 11356 | 16470 |
|  | Unvaried sites | 965 | 1119 | 2533 | 1495 | 11133 | 16187 |
| Island Only | Number of varied sites | 11 | 3 | 3 | 6 | 51 | 68 |
|  | Proportion of varied sites | 0.0111 | 0.0026 | 0.0012 | 0.0040 | 0.0045 | 0.0041 |
| Mainland Only | Number of varied sites | 21 | 18 | 11 | 12 | 175 | 219 |
|  | Proportion of varied sites | 0.0212 | 0.0158 | 0.0043 | 0.0079 | 0.0154 | 0.0133 |
| Island and Mainland | Number of varied sites | 27 | 21 | 14 | 18 | 223 | 283 |
|  | Proportion of varied sites | 0.0272 | 0.0184 | 0.0055 | 0.0119 | 0.0196 | 0.0172 |
|  | Transitions^a^ | 26 | 19 | 13 | 18 | 216 | 274 |
|  | Transversions^a^ | 1 | 2 | 1 | 0 | 9 | 11 |
|  | Transition/transversion ratio | 26 | 9.5 | 13 | - | 24 | 24.91 |
|  | Haplotypes Recovered | 20 | 15 | 13 | 16 | 33 | 35 |

^a^ Calculated in Arlequin v.3.5
