# Supplementary material for: Mitochondrial Genomes Suggest Rapid Evolution of Dwarf California Channel Islands Foxes (Urocyon littoralis)
Source: PLoS One. 2015 Feb 25;10(2):e0118240. doi: 10.1371/journal.pone.0118240 (PMC4340941; doi:10.1371/journal.pone.0118240)
Supplement: S3 Table — (DOCX) [file pone.0118240.s007.docx]

Table S3: AMS Radiocarbon dates of island foxes

| **Locality** | | **Collection** | **AMS Lab Number** | **Material Dated** | **δ13C ‰ VPDB** | **Conventional radiocarbon age, 14C yr BP ± 1 SD** | **cal yr BP age range (2 sigma)** | **Source** |
| --- | --- | --- | --- | --- | --- | --- | --- | --- |
| San Miguel Island | Locality V-7c | SBMNH | UCIAMS-40173 | Bone Collagen | −17.0 | 6120 ± 25 | 7160-6910 | Rick et al 2009 |
|  | Locality V-10c | SBMNH | UCIAMS- 38253 | Bone Collagen | −18.4 | 990 ± 15 | 950–800 | Rick et al 2009 |
|  | Locality V-11 | SBMNH | UCIAMS- 38252 | Bone Collagen | −19.5 | 220 ± 15 | 300–0 | Rick et al 2009 |
| Santa Rosa Island | Upper Tecolote Member | SBMNH | UCR-3563 | Bone Collagen | n/a | 1440 ± 50 | 1510–1280 | Shelley 2001 |
|  | SCRI-333 | UCSB-Repository | OxA-29196 | Bone Collagen | -17.38 | 5290 ± 30 | 6180-5955 | This Paper |
| Santa Cruz Island |  |  |  |  |  |  |  |  |
| Catalina Island | SCAI-17 Pit7 | Catalina Island Museum | OxA-27377 | Bone Collagen | -17.64 | 4636 ±28 | 5460-5310 | This Paper |
|  | SCAI-17 Pit 11 | Catalina Island Museum | OxA-27378 | Bone Collagen | -10.89 | 5463 ±30 | 5470-5640* | This Paper |
| San Nicolas Island | SNI-11 | SBMNH | Beta 106185 | Bone Collagen | n/a | 4940 ± 50 | 5070-4790*^ŧ^ | Shelley 2001 |
| San Clemente Island | SCLI-43C | CalState-Northridge | Beta 106185 | Bone Collagen | n/a | 2110 ±50 | 2300-1950 | Shelley 2001 |

* Indicates marine calibration using the Marine13 calibration curve and a local marine correction (delta-R) of 261± 21. ^ŧ^ This sample may be older without a marine correction.
