# Supplementary material for: Mitochondrial Genomes Suggest Rapid Evolution of Dwarf California Channel Islands Foxes (Urocyon littoralis)
Source: PLoS One. 2015 Feb 25;10(2):e0118240. doi: 10.1371/journal.pone.0118240 (PMC4340941; doi:10.1371/journal.pone.0118240)
Supplement: S4 Table — (DOCX) [file pone.0118240.s008.docx]

Table S4. Primer sequences

| Primer | Sequence |
| --- | --- |
| 30240A_L | CATACCCCGAAAATGTTGGT |
| 30240A_R | GGCGATGGAGGAGTATGCTA |
| 30240B_L | CTGAAATTTGCGGATCCAAC |
| 30240B_R | GGCCGAGCAGATTAGTTGAG |
| 30240C_L | ACGACTGAATGCAGGGCTAT |
| 30240C_R | TGCGACTATGGATTCGTTCA |
| 30240D_L | TTATCCATGGGCCAAAAATC |
| 30240D_R | TAAGCTTTGTGGGCTTTGCT |
| 30240E_L | AACATGAATCGGAGGTCAGC |
| 30240E_R | TGTGTGATCATGGGCTGATT |
| M496L | CGAAGAATCCCGAACTCAAA |
| M496R | TAGGCTTGAATCAGGGCAAC |
| M496_2R | GCATCATACCCTCGATTCCG |
| FMitoF | CGAAGAATCCCGAACTCAAA |
| FMitoR | ATGGGTTTGGTGGGTCATTA |
