# Supplementary material for: Mitochondrial Genomes Suggest Rapid Evolution of Dwarf California Channel Islands Foxes (Urocyon littoralis)
Source: PLoS One. 2015 Feb 25;10(2):e0118240. doi: 10.1371/journal.pone.0118240 (PMC4340941; doi:10.1371/journal.pone.0118240)
Supplement: S5 Table — (DOCX) [file pone.0118240.s009.docx]

Table S5 Distances between Mainland and Islands

| **Locality** | **Distance from the mainland (km)^a^** | **Distance to next closest island with foxes (km)** | **Area (km^2^)** | **2012**  **Estimated Population^b^** |
| --- | --- | --- | --- | --- |
| San Miguel | 42 | 5 | 37 | 538 |
| Santa Rosa | 44 | 5 | 217 | 637 |
| Santa Cruz | 30 | 9 | 249 | 1354 |
| Santa Catalina | 32 | 34 | 194 | 1502 |
| San Nicolas | 98 | 77 | 58 | No Estimate |
| San Clemente | 79 | 34 | 145 | 795 |

^a^Physical characteristics based on Schoenherr et al. (1999:7).

^b^Estimates based spatially-explicit capture-recapture models and includes adults and pups (Island Fox Recovery Meeting 2013)
