# Supplementary material for: Mitochondrial Genomes Suggest Rapid Evolution of Dwarf California Channel Islands Foxes (Urocyon littoralis)
Source: PLoS One. 2015 Feb 25;10(2):e0118240. doi: 10.1371/journal.pone.0118240 (PMC4340941; doi:10.1371/journal.pone.0118240)
Supplement: S1 Text — (DOCX) [file pone.0118240.s010.docx]

**Text S1. Supplementary Materials**

**Radiocarbon dating**

To help contextualize our genetic data, we obtained radiocarbon dates on three island fox bones (identified through comparative zooarchaeological analysis) from Santa Cruz and Santa Catalina islands (Table S3). Previous ^14^C analyses of six fox bones from San Miguel, Santa Rosa, San Nicolas, and San Clemente islands have demonstrated that the oldest island fox bones from the Channel Islands come from subfossil and archaeological deposits dated to 7160 cal BP or younger [1,2]. The three new ^14^C dates we report help expand the geographic coverage of island fox ^14^C ages.

For the three new ^14^C dates reported here, we removed a small (ca. 1000 mg) fragment of island fox bone using a clean blade on a Dremel tool. These bone fragments, were then sent to the Oxford Radiocarbon Accelerator Unit at the University of Oxford. The bones were pretreated using ultrafiltration techniques and collagen was extracted and analyzed for the ^14^C date. For additional details see: <http://c14.arch.ox.ac.uk/embed.php?File=index.html>.

All dates were calibrated using OxCal v. 4.2 [3,4]. Because some foxes may have been consuming high amounts of marine resources, which would require a reservoir correction, the ORAU obtained the ^13^C values for each specimen independently from the radiocarbon analysis. δ^13^C values above –11 were given a marine correction[1]. One specimen required a **Δ**R correction (261 ±21;[5]) and all dates were calibrated using the Intcal13 or Marine13 calibration datasets [6].

Collectively, these data support previous studies, which demonstrated that all known island fox bones post-date human colonization of the Channel Islands (~13,000 cal BP) by a few millennia [1]. The oldest date is from San Miguel at ca. 7160 cal BP, with new dates from Santa Cruz at ~6000 cal BP and Catalina at ~5600 cal BP. Whiles these bones are likely not the oldest island foxes, they support a fox arrival after human colonization and provide an independent means to evaluate our genetic estimates of colonization and divergence.

**Mitochondrial genomes**

185 complete mitochondrial genomes were sequenced from 201 blood and tissue samples (Table S1) extracted using the DNEasy Blood and Tissue DNA kits (Qiagen). Sequencing libraries were prepared with three different protocols on two platforms and identical haplotypes were recovered between platforms indicating minimal bias. The only discrepancy between the three library prep methods was primer bias in samples that were amplified using long-range PCR. These conserved sites were corrected for downstream analysis.

**454 Sequencing**

Using two sets of primers developed by Sasaki et al. (2005), for 16 samples, long-range PCR products of 7-9 kb were amplified in 25 ul reactions containing 1X LA PCR Buffer (TaKaRa), 0.5 mM MgCl_2_, 0.4 mM dNTPs, 0.4 mM of each primer, 1.25 U of TaKaRa LA Hot Start Taq (TaKaRa) [7]. Long-range PCR products for each sample were quantified by spectrophotometry (NanoDrop v 2.0) and pooled in equimolar ratios to 500-1500 ng and were sheared on the QSonica Q800R sonicator for one minute (15 seconds on and 15 seconds off, 25% amplitude) to roughly 400 bp. The pooled sheared sample was prepared for 454 sequencing following magnetic bead purification with an AMPure substitute (2x) (subsequently called SpeedBeads) and eluted in 15ul of ddH20 [8]. Beads were left in the reaction and PEG solution was subsequently used to purify the libraries. Libraries were prepared with a 25 ul blunt end repair reaction of 1X Quick Blunting Buffer (New England Biolabs), 0.1 mM dNTPs, 1ul Quick Blunting Enzyme mix (New England Biolabs). Reactions were incubated at room temperature for 30 minutes and heat inactivated at 70 for 10 minutes. Adapters with individual barcodes were ligated in 54.5 ul reactions of 1x Quick Ligation Buffer (New England Biolabs), 7.4 uM of Adapter A and Adapter B, and 2.5 ul of Quick Ligase enzyme mix (New England Biolabs) [9]. Reactions were incubated at room temperature for 20 minutes, cleaned with 2x PEG solution and eluted in 15ul of ddH20. Adapter fill-in was completed in 30 ul reactions with 1X ThermoPol Buffer, 0.25mM dNTPs and 8 U Bst Polymerase and heated to 37 C for 20 minutes and 70 C for 10 minutes. Reactions were cleaned with 2x PEG solution, eluted in 15 ul of ddH20 and amplified with emulsion PCR primers. Libraries were amplified in 50 ul reactions with 1X Phusion High Fidelity PCR Master Mix with HF buffer (New England Biolabs) and 0.6 mM of each forward and reverse primer. Cycling conditions were as follows 98.0**°**C for 30 seconds, 15 cycles of 98.0**°**C for 10 seconds, 60.0**°**C for 20 seconds, 72.0**°**C for 50 seconds and a final extension of 72.0**°**C for 4 min. Libraries were cleaned 2x PEG solution and gel extracted in 1.5% agarose gel and MiniElute Gel Extraction Kit (Qiagen). Libraries were quantified using 10 ul reactions of 454 Library Quantification kits (Kapa) and were sequenced on one run of the 454 Jr. (Roche). The data was demultiplexed using 454 software and quality filtered using PrinSeq-lite v0.20.3 [10]. Reads were trimmed and filtered until the mean read quality score was above 20. Three 454 libraries were combined and assembled *de novo* using Mira v3.4.0 to generate a reference of 16,718 bp[11]. The contigs were aligned with MAFFT v7.017 to the red fox and dog genome to generate a complete gray fox mitogenome reference in Geneious v.5.6.4 [12–14].

**Long-Range PCR and Illumina sequencing**

An additional 55 samples were prepared for Illumina sequencing following long-range PCR as described above. Long-range PCR products were pooled to 500ng in equimolar rations and sheared using QSonica Q800R sonicator for two minute (15 seconds on and 15 seconds off, 25% amplitude) to roughly 600 bp and libraries were prepared with iNext dual indexed adapters [15] by cleaning with 2.5X SpeedBeads. The ends of sheared pooled samples were repaired in 48 ul reactions with 1X NEB Buffer 2, 0.03 mM dNTPs, and 1.8 U DNA Polymerase I, Large (Klenow) Fragment (New England Biolabs) and incubated for 15 minutes at 25.0**°**C and 20 minutes at 75.0**°**C. Samples were cleaned with 2.5X PEG solution and eluted in 25 ul of ddH20. Instead of d-A tailing, we made dC buffer by adding dCTP (Fermentas) to 10x NEB Buffer for a final dCTP concentration of 2mM. d-C tailing 50 ul reaction consisted of 1x NEB Buffer 2 + dCTP, 15 U Klenow Fragment (3’-5’ exo) (New England Biolabs) and were incubated at 37.0**°**C for 30 minutes. Following dC-tailing, libraries were cleaned with 2X PEG solution, eluted in 25 ul of ddH20. A stubby adapter was ligated in 50 ul reactions with the NEBNext Quick Ligation kit following the manufacturers instructions with 0.1 uM of stubby adapter and incubated for 15 min at 20.0**°**C and 10 minutes at 65.0**°**C. The reactions were cleaned with 2x PEG solution and suspended in 25 ul ddH20. Each sample was amplified in 50 ul reactions of 1x Kapa HiFI Hot Start ReadyMix, 0.5 uM of each i5 and i7 indexing primer and 10 ul of ligated sample. Cycling conditions were 45 seconds at 98.0**°**C, 15 cycles of 15 seconds at 98.0**°**C, 30 seconds at 60.0**°**C and 30 seconds at 72.0**°**C, followed by 3 minutes at 72. Libraries were cleaned with 2X PEG solution, eluted in 25 ul of ddH20 and quantified using 10 ul reactions of the Illumina Library Quantification kit (Kapa). Libraries were pooled in equimolar ratios for sequencing in one 100 BP paired-end Illumina HiSeq lane. Reads were demultiplexed allowing for one mismatch using CASAVA v1.8.0.

**Capture and Illumina sequencing**

Based on the 454 dataset, 658 80bp probes with 2x tiling were designed to capture the variation in nine island and mainland mitogenomes generated from 454 data. The RNA probes were synthesized in the MyBaits-1 kit (Mycroarray) for in-solution capture (MitogenomeProbes.fa). Genomic DNA was sheared using QSonica Q800R sonicator for 2 minutes 15 seconds (15 seconds on and 15 seconds off, 25% amplitude) to roughly 600 bp and 130 libraries were prepared with Nextera-style dual indexed adapters as described above. Libraries were pooled in groups of 6-8 individuals and captured for 24 hours following the manufacturer’s protocol and were eluted in 30 ul of ddH20. Post-capture the libraries were amplified in 50 ul reactions with 1x Kapa HiFI Hot Start ReadyMix, 0.5 uM of each Illumina primer and 10 ul of captured sample. Cycling conditions were 45 seconds at 98.0**°**C, 10-16 cycles of 15 seconds at 98.0**°**C, 30 seconds at 60.0**°**C and 30 seconds at 72.0**°**C, followed by 3 minutes at 72. Libraries were quantified using 10 ul reactions of the Illumina Library Quantification kit (Kapa) and pooled in equimolar ratios for sequencing in one 100 BP paired-end Illumina HiSeq lane. Reads were demultiplexed allowing for one mismatch using CASAVA v1.8.0.

**Data filtering and assembly**

All 454 and Illumina data were trimmed and quality filtered using the PrinSeq-lite v0.20.3 so that the mean read quality was above 20 on the phred scale. Filtered data was mapped with BWA v.0.7.4 to the gray fox reference and a consensus sequence and coverage information were generated using SamTools v0.1.19 [16,17]. Alignments with ambiguous bases were visually examined and all samples with missing data (n= 14) were removed. An additional two samples were removed following Sanger sequencing.

Consensus sequences for each individual were aligned with Mafft v7.017 as implemented in Geneious 7.06 [12,13]. A highly repetitive region in the control region was deleted in all samples due to mapping and assembly problems with repetitive runs. The resulting alignment of 185 fox mitogenomes of 16,470 bp each totals over 3 million basepairs.

**Sanger sequencing verification**

For ambiguous sites and haplotypes represented by a single individual, 8 pairs of primers (Table S4) were designed in Primer3 to confirm the base call [18]. PCR reactions were done in 25 ul reactions of 1X Gold buffer (Perkin-Elmer, ABI), 0.2 mM of dNTPs, 2 mM MgCl_2_, 0.4 mM of each primer, 0.8 mg/ml BSA (New England Biolabs) and (1 U of Taq Gold (Perkin-Elmer, ABI). Cycling conditions were 10 minutes at 95.0**°**C, and 35 cycles of 1 minute at 94.0**°**C, 1 minute at 50.0**°**C, and 1 minute at 72.0**°**C, with an extension of ten minutes at 72.0**°**C. PCR products were visualized on a 2% agarose gel and excess primers and dNTPs were removed by treatment with 1:10 dilution of ExoSAP-IT (Affymetrix) and six microliters of PCR products and heated to 37°C for 15 min and 85°C for 15 min. Cleaned PCR products were used for cycle sequencing using BigDye terminator premix version 3.1 (Applied Biosystems). Each reaction contained 0.7 μL Big Dye Terminator, 1.5 μl Big Dye Buffer, 5.5 μl PCR grade ddH2O, 0.3uM primer and 2 μl of PCR product. Cycling conditions were 2 minutes at 96.0**°**C, 25 cycles of 96.0**°**C for 10 seconds, 50.0**°**C for 10 seconds and 60.0**°**C for 4 minutes. Reactions were cleaned with Sephadex G-50 fine powder (GE Healthcare) and durapore membrane multiscreen filter plates (Millipore). Products were Sanger sequenced on an ABI 3130xl automated capillary sequencer and Geneious v7.0.6 was used to remove primers and align sequences. Eleven samples were Sanger sequenced (including samples with unambiguous base calls), two samples were thrown out for conflicting Sanger data but all other Sanger data clarified and confirmed our results.

**Phylogeography**

Haplotype diversity, nucleotide diversity and pairwise Fst were calculated in DNAsp v5.10.1 [19]. We found a positive correlation between island area and the number of haplotypes recovered and haplotype diversity (Pearson’s r=0.80 p-value=0.03 and r=0.77 p-value=0.04, respectively) (Figure S3). Number of haplotypes, haplotype diversity, and nucleotide diversity per island were not correlated with distance from the mainland or distance to the closest island with foxes (Table S5). There was an east-west trend in the distribution of genetic variability across the northern islands, with the highest levels of variability in the east (Santa Cruz Island- five haplotypes) and the lowest in the west (San Miguel Island- one haplotype). Arelquin v3.5 was used to calculate the transitions and transversion found in each population [20].

*Network analysis:* Network analysis was conducted on an alignment stripped of monomorphic sites using the median joining algorithm as implemented in program Network v.4.612 using the default parameters [21,22]. Additional networks were generated for just the cytochrome b and d-loop regions (Figure S1). Analysis of cytochrome b (Figure S1B) suggests that a mainland northern California gray fox was introduced to Santa Catalina and later moved to San Clemente, to San Nicolas and to the Northern islands, potentially while the northern islands were connected in Santarosae as they share a single haplotype. The D-loop network (Figure S1B) does better that cytb in recovering unique haplotypes, but the region still on recovers only 20 haplotypes. These networks suggest a very different population structure and evolutionary history than complete mitogenomes.

**Selection analysis**

To test for selection, an alignment of representative haplotypes was curated for coding genes only. As the mitogenome is a single unit of inheritance without recombination, the alignment was not partitioned in this analysis. Regions of genes with overlapping frames with another gene (ATP6/ATP8), were duplicated to allow for independent selection on overlapping codons. NADH6, which is coded on the opposite strand, was reverse complemented in the alignment to allow for a single reading frame across the coding genes. Stop codons were removed resulting in alignment of 11,286 bp. The HKY85 model was used for all subsequent selection analysis. We conducted selection analyses using six algorithms (SLAC, REL, FEL, IFEL, MEME, FUBAR) to test for mitogenome wide selection, codon specific selection and episodic diversifying selection [23–27]. Codon 258 in NADH1 was identified as under positive selection using FEL, IFEL, MEME and FUBAR, with p-values approaching significant in IFEL (0.07) and under 0.2 in FEL and MEME. The posterior probability for FUBAR was 0.855. PROVEAN v1.1.3 was used to determine if the changes in codon 258 affected chemical properties of the protein [28,29]. With a PROVEAN score of 0.096 (cutoff -2.5), this substitution is predicted to be neutral.

**Phylogenetic analysis**

To examine phylogenetic relationships between island, California and eastern gray foxes, additional publically available mammal mitochondrial genomes were obtained from GenBank and aligned to the fox dataset using Mafft v7.017 as implemented in Geneious 7.06[12,13]. The alignment was run through jModelTest v.2.1 and the GTR+I+G model was used to run 1000 pseudobootstrap replicates of the maximium likelihood tree program Garli (Figure S2) as implemented on the Lattice grid computing system [30–35]. We also conducted a parsimony analysis in PAUP* v4.0a131 that yielded the same topology as the maximum likelihood and Bayesian analyses.

To date the divergence between island and mainland foxes, Bayesian phylogenetic analysis was conducted in BEAST v.1.7.5 as implemented on the CIPRES web portal [36–38]. The eastern gray fox was used as an outgroup as indicated by the maximum likelihood analysis. Each gene was run through JModelTest v2.1 separately as well as the entire alignment and PartitionFinder v1.1.1 was used to test for codon partitioning. Based on this analysis, no codon partitioning and empirical base frequencies were used with each gene fitting the HKY or the TN93 model. We tested for a strict molecular clock in MEGA5 [39] and equal evolutionary rates were rejected for both GTR and HKY models. However both a strict and a lognormal relaxed clock were used with a coalescent of constant size tree prior. The earliest radiocarbon date was used as a prior as the time to the most recent common ancestor for all island samples with a normal distribution around the mean of 0.0070 (quantiles: median: 7E-3, 2.5% 5.04E-3, 97.5%: 8.96E-3) and standard deviation of 0.0010. The ucld.mean was changed to gamma distribution initial value 1, shape 0.0010, scale 1000 offset 0. As the eastern gray fox was the outgroup in this analysis, we set the tree model root length to the early Pliocene *Urocyon* fossil dating to 5.332-2.558 MYA (lognormal distribution 2.5% 0.273, median: 1.941, 97.5%: 13.78) [40]. The root height was set with a lognormal distribution and initial value of 2.2, mean 3.2 with a log(stdev) of 1. All other priors were left to default settings and the MCMC was run in two independent runs of 100 million chains each, logging every 10,000 chains. The log files were examined in Tracer v1.6 to examine for convergence [41]. An empty alignment was tested to sample for effects of the prior and the resulting poor posterior and prior ESS with values below 200 indicated that the priors were not strongly influencing the tree.

The mean substitution rate estimated in this analysis was 9.83% (95%HPD 5.557-14.52) per million years with a standard deviation of 2.35% and a median of 9.57% per million years for the run assuming a relaxed molecular clock. We compared this with the rates calculated from a strict molecular clock, which were 10 % per million years with a standard deviation of 2.35% and a median of 10% per million years. The strict clock was also tested even though the molecular clock test rejected, possibly as a result of serial bottlenecks in island foxes. Regardless, the rates are very similar, and do not effect the overall results of this analysis. We surveyed the literature for canid and mammal substitution rates and developed a database of rates for different taxa and markers [42–53]. Our estimated rates fall within canid substitution rates, which vary between markers, between taxa pairs and depending on which fossil calibration was used. Rates for human mitogenomes vary between 6.8 and 9.66 per site per million year depending on whether chimps are included or not included and in dog-wolf-coyotes between 0.64 and 1.92 per site per million year with an average of 3.3 per site per million years for mammals [42–53]. The calculated substitution rate may be higher than expected due to the recent bottleneck due to a distemper outbreak in Channel Island foxes [54]. This could explain why the substitution rates are clustering beyond the average mitochondrial genome rates for a large dataset of mammals. Due to the distance between gray foxes and other canids, estimated to be greater than 10 million years [55], including an external calibration point is difficult when examining very shallow divergences. Comparison of small regions of the mitogenome to other canid datasets is not possible as there is not enough variation in these regions to resolve island fox biogeography. All of these issues lead to the elevated rate estimated and is a known problem in recent divergences [56,57].

**References**

1. Rick TC, Erlandson JM, Vellanoweth R, Braje TJ, Guthrie DA, et al. (2009) Origins and Antiquity of the Island Fox (Urocyon littoralis) on California’s Channel Islands. Quat Res 71: 93–98.

2. Shelley SD (2001) Archaeological Evidence of the Island Fox (Urocyon littoralis) on California’s Channel Islands. Prepared for Naval Air Weapons Station, Point Mugu.

3. Ramsey CB (2009) Bayesian Analysis of Radiocarbon Dates. Radiocarbon 51: 337–360. doi:10.2458/azu_js_rc.51.3494.

4. Ramsey CB (2013) OxCal 4.2. Available: http://c14.arch.ox.ac.uk/.

5. Jazwa C, Kennett D, Hanson D (2012) Late Holocene Subsistence Change and Marine Productivity on Western Santa Rosa Island, Alta California. Calif Archaeol 4: 69–98. doi:10.1179/cal.2012.4.1.69.

6. Reimer P (2013) IntCal13 and Marine13 Radiocarbon Age Calibration Curves 0–50,000 Years cal BP. Radiocarbon 55: 1869–1887. doi:10.2458/azu_js_rc.55.16947.

7. Sasaki T, Nikaido M, Hamilton H, Goto M, Kato H, et al. (2005) Mitochondrial Phylogenetics and Evolution of Mysticete Whales. Syst Biol 54: 77–90. doi:10.1080/10635150590905939.

8. Rohland N, Reich D (2012) Cost-effective, high-throughput DNA sequencing libraries for multiplexed target capture. Genome Res: gr.128124.111. doi:10.1101/gr.128124.111.

9. Meyer M, Stenzel U, Hofreiter M (2008) Parallel tagged sequencing on the 454 platform. Nat Protoc 3: 267–278. doi:10.1038/nprot.2007.520.

10. Schmieder R, Edwards R (2011) Quality control and preprocessing of metagenomic datasets. Bioinforma Oxf Engl 27: 863–864. doi:10.1093/bioinformatics/btr026.

11. Chevreux B, Wetter T, Suhai S (1999) Genome Sequence Assembly Using Trace Signals and Additional Sequence Information. Ger Conf Bioinforma. Available: http://citeseerx.ist.psu.edu/viewdoc/summary?doi=10.1.1.23.7465. Accessed 13 December 2012.

12. Geneious (n.d.). Biomatters. Available: http://www.geneious.com/​​.

13. Katoh K, Misawa K, Kuma K, Miyata T (2002) MAFFT: a novel method for rapid multiple sequence alignment based on fast Fourier transform. Nucleic Acids Res 30: 3059–3066. doi:10.1093/nar/gkf436.

14. Katoh K, Standley DM (2013) MAFFT Multiple Sequence Alignment Software Version 7: Improvements in Performance and Usability. Mol Biol Evol 30: 772–780. doi:10.1093/molbev/mst010.

15. Glenn TC, Nilsen R, Kieran TJ, Finger Jr. JW, Pierson TW, et al. (n.d.) Adapterama I: Universal stubs and primers for thousands of dual-indexed Illumina Nextera and TruSeqHT compatible libraries (iNext & iTru). Be Submitted Mol Ecol Resour.

16. Li H, Durbin R (2010) Fast and accurate long-read alignment with Burrows-Wheeler transform. Bioinforma Oxf Engl 26: 589–595. doi:10.1093/bioinformatics/btp698.

17. Li H, Handsaker B, Wysoker A, Fennell T, Ruan J, et al. (2009) The Sequence Alignment/Map format and SAMtools. Bioinforma Oxf Engl 25: 2078–2079. doi:10.1093/bioinformatics/btp352.

18. Rozen S, Skaletsky HJ (1998) Primer3. Available: Code available at http://www-genome.wi.mit.edu/genome_software/other/primer3.html.

19. Librado P, Rozas J (2009) DnaSP v5: a software for comprehensive analysis of DNA polymorphism data. Bioinformatics 25: 1451–1452. doi:10.1093/bioinformatics/btp187.

20. Excoffier L, Lischer HEL (2010) Arlequin suite ver 3.5: a new series of programs to perform population genetics analyses under Linux and Windows. Mol Ecol Resour 10: 564–567. doi:10.1111/j.1755-0998.2010.02847.x.

21. Bandelt HJ, Forster P, Röhl A (1999) Median-joining networks for inferring intraspecific phylogenies. Mol Biol Evol 16: 37–48.

22. Network (2014). Available: fluxus-engineering.com.

23. Murrell B, Moola S, Mabona A, Weighill T, Sheward D, et al. (2013) FUBAR : A Fast, Unconstrained Bayesian AppRoximation for inferring selection. Mol Biol Evol: mst030. doi:10.1093/molbev/mst030.

24. Pond SLK, Murrell B, Fourment M, Frost SDW, Delport W, et al. (2011) A random effects branch-site model for detecting episodic diversifying selection. Mol Biol Evol: msr125. doi:10.1093/molbev/msr125.

25. Kosakovsky Pond SL, Frost SDW, Grossman Z, Gravenor MB, Richman DD, et al. (2006) Adaptation to Different Human Populations by HIV-1 Revealed by Codon-Based Analyses. PLoS Comput Biol 2: e62. doi:10.1371/journal.pcbi.0020062.

26. Murrell B, Wertheim JO, Moola S, Weighill T, Scheffler K, et al. (2012) Detecting individual sites subject to episodic diversifying selection. PLoS Genet 8: e1002764. doi:10.1371/journal.pgen.1002764.

27. Pond SLK, Frost SDW (2005) Not So Different After All: A Comparison of Methods for Detecting Amino Acid Sites Under Selection. Mol Biol Evol 22: 1208–1222. doi:10.1093/molbev/msi105.

28. Choi Y (2012) A Fast Computation of Pairwise Sequence Alignment Scores Between a Protein and a Set of Single-locus Variants of Another Protein. Proceedings of the ACM Conference on Bioinformatics, Computational Biology and Biomedicine. BCB ’12. New York, NY, USA: ACM. pp. 414–417. Available: http://doi.acm.org/10.1145/2382936.2382989. Accessed 19 February 2014.

29. Choi Y, Sims GE, Murphy S, Miller JR, Chan AP (2012) Predicting the Functional Effect of Amino Acid Substitutions and Indels. PLoS ONE 7: e46688. doi:10.1371/journal.pone.0046688.

30. Bazinet AL, Cummings MP (2011) Computing the Tree of Life: Leveraging the Power of Desktop and Service Grids. Proceedings of the 2011 IEEE International Symposium on Parallel and Distributed Processing Workshops and PhD Forum. IPDPSW ’11. Washington, DC, USA: IEEE Computer Society. pp. 1896–1902. Available: http://dx.doi.org/10.1109/IPDPS.2011.344. Accessed 13 December 2012.

31. Bazinet A, Myers D, Fuetsch J, Cummings M (2007) Grid Services Base Library: A high-level, procedural application programming interface for writing Globus-based Grid services. Future Gener Comput Syst 23: 517–522. doi:10.1016/j.future.2006.07.009.

32. Bazinet A, Cummings MP (2008) Distributed & Grid Computing –- Science Made Transparent for Everyone. Principles, Applications and Supporting Communities. Marburg: Rechenkraft.net. 2-13 p.

33. Cummings MP, Handley SA, Myers DS, Reed DL, Rokas A, et al. (2003) Comparing bootstrap and posterior probability values in the four-taxon case. Syst Biol 52: 477–487.

34. Zwickl D (2006) Genetic algorithm approaches for the phylogenetic analysis of large biological sequence datasets under the maximum likelihood criterion.

35. Darriba D, Taboada GL, Doallo R, Posada D (2012) jModelTest 2: more models, new heuristics and parallel computing. Nat Methods 9: 772–772. doi:10.1038/nmeth.2109.

36. Miller MA, Pfeiffer W, Schwartz T (2010) Creating the CIPRES Science Gateway for Inference of Large Phylogenetic Trees. Proceedings of the Gateway Computing Environments Workshop (GCE). New Orleans, LA. pp. 1–8.

37. Drummond AJ, Suchard MA, Xie D, Rambaut A (2012) Bayesian Phylogenetics with BEAUti and the BEAST 1.7. Mol Biol Evol. Available: http://mbe.oxfordjournals.org/content/early/2012/02/25/molbev.mss075. Accessed 3 May 2012.

38. Drummond AJ, Rambaut A (2007) BEAST: Bayesian evolutionary analysis by sampling trees. BMC Evol Biol 7: 214. doi:10.1186/1471-2148-7-214.

39. Tamura K, Peterson D, Peterson N, Stecher G, Nei M, et al. (2011) MEGA5: molecular evolutionary genetics analysis using maximum likelihood, evolutionary distance, and maximum parsimony methods. Mol Biol Evol 28: 2731–2739. doi:10.1093/molbev/msr121.

40. McKenna MC, Bell SK (1997) Classification of Mammals: Above the Species Level. Columbia University Press. 652 p.

41. Rambaut A, Suchard MA, Xie D, Drummond AJ (2013) Tracer v1.5. Available: Available from http://beast.bio.ed.ac.uk/Tracer.

42. Nabholz B, Glémin S, Galtier N (2008) Strong Variations of Mitochondrial Mutation Rate across Mammals—the Longevity Hypothesis. Mol Biol Evol 25: 120–130. doi:10.1093/molbev/msm248.

43. Kutschera VE, Lecomte N, Janke A, Selva N, Sokolov AA, et al. (2013) A range-wide synthesis and timeline for phylogeographic events in the red fox (Vulpes vulpes). BMC Evol Biol 13: 114. doi:10.1186/1471-2148-13-114.

44. Edwards CJ, Soulsbury CD, Statham MJ, Ho SYW, Wall D, et al. (2012) Temporal genetic variation of the red fox, Vulpes vulpes, across western Europe and the British Isles. Quat Sci Rev 57: 95–104. doi:10.1016/j.quascirev.2012.10.010.

45. Freedman AH, Gronau I, Schweizer RM, Ortega-Del Vecchyo D, Han E, et al. (2014) Genome Sequencing Highlights the Dynamic Early History of Dogs. PLoS Genet 10: e1004016. doi:10.1371/journal.pgen.1004016.

46. Endicott P, Ho SYW (2008) A Bayesian Evaluation of Human Mitochondrial Substitution Rates. Am J Hum Genet 82: 895–902. doi:10.1016/j.ajhg.2008.01.019.

47. Heller R, Brüniche-Olsen A, Siegismund HR (2012) Cape buffalo mitogenomics reveals a Holocene shift in the African human–megafauna dynamics. Mol Ecol 21: 3947–3959. doi:10.1111/j.1365-294X.2012.05671.x.

48. Savolainen P, Zhang Y, Luo J, Lundeberg J, Leitner T (2002) Genetic Evidence for an East Asian Origin of Domestic Dogs. Science 298: 1610–1613. doi:10.1126/science.1073906.

49. Dalén L, Fuglei E, Hersteinsson P, Kapel CMO, Roth JD, et al. (2005) Population history and genetic structure of a circumpolar species: the arctic fox. Biol J Linn Soc 84: 79–89. doi:10.1111/j.1095-8312.2005.00415.x.

50. Pang J-F, Kluetsch C, Zou X-J, Zhang A, Luo L-Y, et al. (2009) mtDNA Data Indicate a Single Origin for Dogs South of Yangtze River, Less Than 16,300 Years Ago, from Numerous Wolves. Mol Biol Evol 26: 2849–2864. doi:10.1093/molbev/msp195.

51. Sacks BN, Louie S (2008) Using the dog genome to find single nucleotide polymorphisms in red foxes and other distantly related members of the Canidae. Mol Ecol Resour 8: 35–49. doi:10.1111/j.1471-8286.2007.01830.x.

52. Bardeleben C, Moore RL, Wayne RK (2005) Isolation and Molecular Evolution of the Selenocysteine tRNA (Cf TRSP) and RNase P RNA (Cf RPPH1) Genes in the Dog Family, Canidae. Mol Biol Evol 22: 347–359. doi:10.1093/molbev/msi022.

53. Vila, Amorim, Leonard, Posada, Castroviejo, et al. (1999) Mitochondrial DNA phylogeography and population history of the grey wolf canis lupus. Mol Ecol 8: 2089–2103.

54. Coonan TJ, Schwemm CA, Garcelon DK (2010) Decline and Recovery of the Island Fox: A Case Study for Population Recovery. Cambridge University Press. 229 p.

55. Lindblad-Toh K, Wade CM, Mikkelsen TS, Jaffe DB, Kamal M, et al. (2005) Genome sequence, comparative analysis and haplotype structure of the domestic dog. Nature 438: 803–819. doi:10.1038/nature04338.

56. Ho SYW, Phillips MJ, Cooper A, Drummond AJ (2005) Time Dependency of Molecular Rate Estimates and Systematic Overestimation of Recent Divergence Times. Mol Biol Evol 22: 1561–1568. doi:10.1093/molbev/msi145.

57. Ho SYW, Shapiro B, Phillips MJ, Cooper A, Drummond AJ (2007) Evidence for Time Dependency of Molecular Rate Estimates. Syst Biol 56: 515–522. doi:10.1080/10635150701435401.

**Figure Legends**

**Figure S1. Network Analysis of cytochrome b and d-loop.** Cytochrome b (1140 bp) only network (A) and d-loop (992 bp) only network (B) were generated from variable alignment sites. The size of the circles is proportional to the number of individuals represented by it. Neither cytochrome b nor d-loop had enough variants to detect all island-specific lineages.

**Figure S2. Maximum Likelihood Tree of Island and Gray Foxes.** Rooted tree generated in Garli with 1000 bootstrap replicates. Key nodes are shown with bootstrap support. Nodes that are not labeled may also have strong support. Eastern gray fox is basal to the California clade and there is strong support for Clade A and Clade B (Figure 3).

**Figure S3. Haplotype and Haplotype Diversity Correlate with Island Area.** A positive correlation was identified between island area and the number of haplotypes recovered and haplotype diversity (Pearson’s r=0.80 p-value=0.03 and r=0.77 p-value=0.04, respectively).
